# A Review of Sharp-Switching Band-Modulation Devices

**DOI:** 10.3390/mi12121540

**Published:** 2021-12-11

**Authors:** Sorin Cristoloveanu, Joris Lacord, Sébastien Martinie, Carlos Navarro, Francisco Gamiz, Jing Wan, Hassan El Dirani, Kyunghwa Lee, Alexander Zaslavsky

**Affiliations:** 1 IMEP-LAHC, Université Grenoble Alpes, Grenoble INP & CNRS, 3 Parvis Louis Néel, CS 50257, CEDEX 1, 38016 Grenoble, France; hassan_dir@hotmail.com (H.E.D.); lkh910928@gmail.com (K.L.); 2 CEA, LETI, MINATEC Campus, Université Grenoble Alpes, 17 Rue des Martyrs, CEDEX 9, 38054 Grenoble, France; Joris.LACORD@cea.fr (J.L.); Sebastien.MARTINIE@cea.fr (S.M.); 3 Nanoelectronics Research Group, CITIC-UGR, University of Granada, 18071 Granada, Spain; carlosnm@ugr.es (C.N.); fgamiz@ugr.es (F.G.); 4 State Key Lab of ASIC and System, School of Information Science and Engineering, Fudan University, Shanghai 200433, China; jingwan@fudan.edu.cn; 5 Department of Physics and School of Engineering, Brown University, Providence, RI 02912, USA; Alexander_Zaslavsky@brown.edu

**Keywords:** nanoelectronics, SOI, FDSOI, electrostatic doping, band modulation, sharp switching, ultrathin body, memory, ESD, sensors, reconfigurable device, Esaki diode

## Abstract

This paper reviews the recently-developed class of band-modulation devices, born from the recent progress in fully-depleted silicon-on-insulator (FD-SOI) and other ultrathin-body technologies, which have enabled the concept of gate-controlled electrostatic doping. In a lateral PIN diode, two additional gates can construct a reconfigurable PNPN structure with unrivalled sharp-switching capability. We describe the implementation, operation, and various applications of these band-modulation devices. Physical and compact models are presented to explain the output and transfer characteristics in both steady-state and transient modes. Not only can band-modulation devices be used for quasi-vertical current switching, but they also show promise for compact capacitorless memories, electrostatic discharge (ESD) protection, sensing, and reconfigurable circuits, while retaining full compatibility with modern silicon processing and standard room-temperature low-voltage operation.

## 1. Introduction

**Figure 1:**
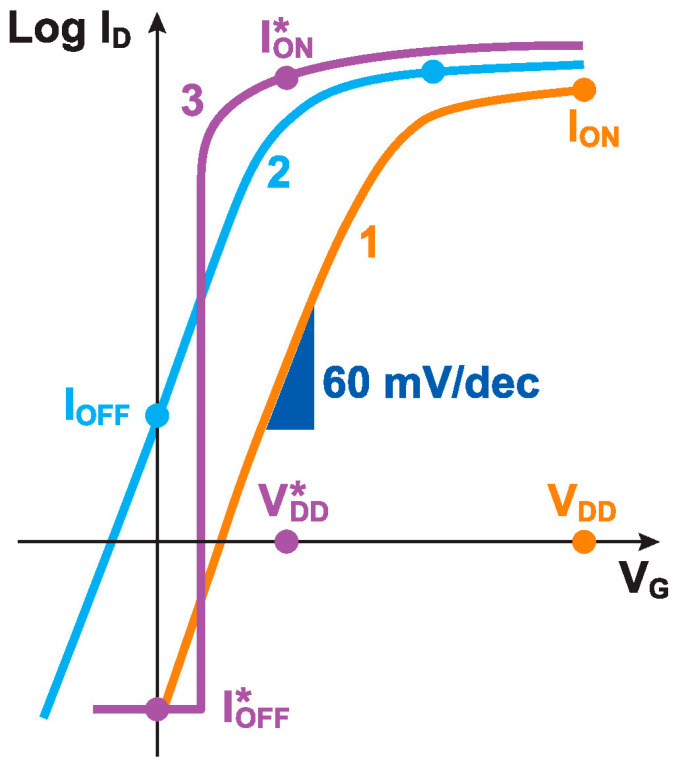
A schematic diagram of the tradeoff between power supply voltage V_DD_ and power consumption. Curve 1 represents an ideal transistor with SS ≈ 60 mV/decade of I_D_ at room temperature. Downscaling the threshold voltage is unavoidable for device miniaturization and power reduction, but this produces curve 2, with an unacceptable I_OFF_. An ideal sharp-switching device (curve 3) would provide the same or higher I*_ON_/I*_OFF_ ratio when operated at a lower V*_DD_.

The search for sharp-switching devices compatible with modern complementary metal-oxide-semiconductor (CMOS) digital technology has been a central preoccupation of the device physics community for the past two decades. With the continuous downscaling of CMOS technology, the switching limit of standard CMOS transistors—the 2.3 kT ≈ 60 mV of the controlling gate voltage V_G_ required to reduce the drain current I_D_ by a decade below the transistor threshold voltage V_T_ at room temperature—is the ultimate obstacle to reducing the supply voltage of large-scale high-performance digital circuitry. This is due to the power consumption issue schematically illustrated in Figure 1. As long as the 60 mV/decade subthreshold slope (SS) limit remains in force, achieving an acceptable I_ON_ for switching downstream transistors and wiring parasitics, while maintaining a sufficiently low I_OFF_ to prevent static power consumption, requires a power supply of roughly V_DD_ ≈ 0.5 V [1]. Reducing the threshold voltage and V_DD_ leads to a catastrophic exponential increase in I_OFF_. Only a hypothetical sharper-switching device exempt from the 60 mV/dec constraint could provide a high I*_ON_ at a low V*_DD_, opening the possibility of many additional generations of low-power downscaled CMOS circuitry.

Physically, the 60 mV/decade limit arises from the fundamental Boltzmann carrier distribution in the source electrode and cannot be overcome in the standard source–gate–drain transistor structure, where V_G_ shuts off the source–drain current by creating a potential barrier in the channel [2]. Whereas various second-order effects that led to even worse SS have been overcome through a sustained research effort into FinFET and fully-depleted SOI (FD-SOI) device technologies [3], true sharp switching requires a modified V_G_-controlled carrier transport mechanism. While there has been no shortage of alternative device candidates, in this review we will restrict ourselves to devices with realistic CMOS integration prospects; even if beyond the current state-of-the-art. We will also limit ourselves to room temperature operation, as cooling standard CMOS to cryogenic temperatures—while certainly providing sharper switching and possibly interesting for applications, such as quantum sensing or computing at sub-1 K dilution temperatures [4,5,6]—does not address the needs of mainstream digital technology, which is evolving towards portable, miniaturized, and, ideally, low-power devices.

With these provisos, a number of fascinating sharp-switching devices will fall outside the scope of this review article. For example, we will not discuss nanoelectromechanical switches based on voltage-controlled gate positioning or even direct physical contact in a cantilever geometry; these devices provide truly sharp switching, but appear unlikely to provide the necessary reliability and immunity to sticking. We will also omit ferroelectric negative capacitance devices, where the hypothetical V_G_ amplification by the polarization in the ferroelectric gate material has not produced experimentally validated sharp switching together with competitively fast operation. The interested reader is referred to other, more complete, sharp-switching device reviews [7]. Instead we will focus on a recently developed class of band modulation devices, where reconfigurable gate-controlled electrostatic doping, combined with multiple gate geometries developed in an industrially-validated FD-SOI process, allow for ultra-sharp switching via a feedback mechanism. These devices have potential applications as logic switches, memories, electrostatic discharge (ESD) protection, and sensing devices; without introducing nonstandard materials, nonstandard processing steps, or cryogenic temperature operation. Furthermore, their reconfigurability enables implementing other popular sharp-switching devices, such as tunneling transistors (TFETs) and impact ionization (I-MOS) transistors, again without abandoning the FD-SOI process.

### 1.1. Electrostatic Doping for Reconfigurable Devices

**Figure 2:**
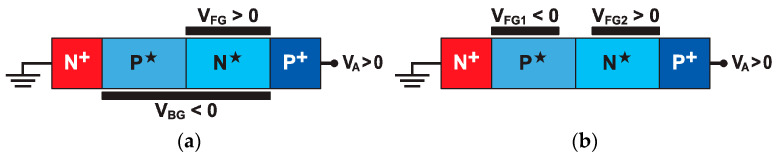
Configuration of band-modulation devices: (**a**) Z^2^–FET fabricated on FD-SOI and (**b**) field-effect diode implementable in nanowires and FinFETs.

Band-modulation devices are virtual PNPN structures with electrostatic doping induced by gate action (Figure 2). Electrostatic doping is a unique asset of modern devices with an ultrathin body layer [8,9,10,11]. A gate voltage attracts free carriers that spread in the entire body, rather than being confined at the semiconductor–dielectric interface, as in bulk-silicon systems. This effect, known as ‘volume inversion’ or ‘volume accumulation’ [3], gives rise to populations of electrons (for V_G_ > 0) or holes (for V_G_ < 0) that behave, in many respects, as those created by conventional physical doping. The tremendous advantage is that the polarity and concentration of such electrostatic doping is adjustable on demand by the gate voltage. The opportunity to emulate P–N junctions in ultrathin fully-depleted semiconductor layers represents a paradigm shift in the design of reconfigurable and multifunctional circuits [11,12].

Consider the FD-SOI device in Figure 2a that combines electrostatic and physical doping. The end contacts, *N*^+^ cathode and *P*^+^ anode, are formed by ion implantation or in situ doping. The undoped body is controlled by a partial front gate (V_FG_) and by the ground-plane acting as a back gate (V_BG_). Each gate can induce P*-type or N*-type electrostatic doping or just leave the gated and ungated regions fully depleted (*I*). The concentration of electrostatically-induced carriers declines from the voltage-controlled interface to the opposite interface. The thinner the body, the more homogeneous the vertical profile of the effective doping.

According to the polarities of the two regions, there are nine possible combinations of the electrostatically-doped body segments, corresponding to P–N diodes, PIN diodes, IMOS, TFET, and PNPN band-modulation transistors. This single structure, which is able to impersonate nine different devices, has received the name ‘hocus-pocus diode’ [12], in order to emphasize the magic of electrostatic doping.

Unlike FD-SOI devices, FinFETs, nanowires, and nanosheets do not enjoy the advantages of a built-in back gate. Nonetheless, the hocus-pocus diode can still be implemented using two adjacent front gates (or surrounding gates), as shown in Figure 2b. In this review, we will mostly focus on the Z^2^–FET variant of Figure 2a, which offers a perfectly sharp junction at the gate edge [13,14].

### 1.2. Principle of Band-Modulation

Although the lateral PNPN structure looks like a floating-body thyristor, its operating principle is different, as it relies on band-modulation effects, rather than impact ionization (Section 3). The device is biased in forward mode with V_A_ > 0. The electrostatic doping builds up three P–N junctions along the body; their energy barriers prevent the injection of holes from the anode and of electrons from the cathode into the body. The device stays in blocked mode, until the gate or anode voltage becomes large enough to collapse the barriers and let a large cathode–anode current flow. Corresponding band diagrams and a detailed description are given in Section 3.1. The switching characteristics are extremely sharp and inspired the name Z^2^–FET, for Zerosubthreshold swing and *Zero* impact ionization [13]. The earlier variant with two top gates, as in Figure 2b, was named a field-effect diode (FED) [15,16].

### 1.3. Device Variants

The architecture of the Z^2^–FET can be further enriched in several ways.

**Figure 3:**
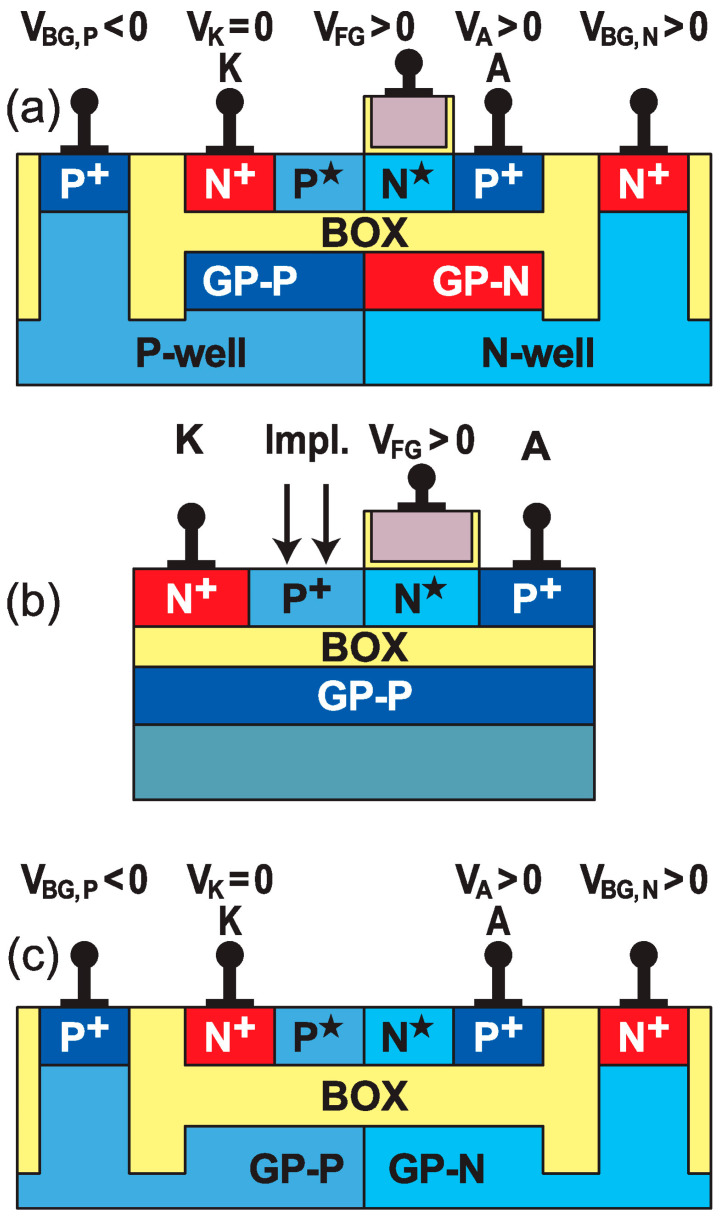
Z^2^–FET variants fabricated with FD-SOI technology: (**a**) dual ground-plane, (**b**) partially doped, and (**c**) gateless Z^3^–FET.

*Dual ground-planes* (Figure 3a): The ground-plane (GP-P) located underneath the ungated portion of the body is negatively biased and generates the necessary P* electrostatic doping. The sister ground-plane, located beneath the gated region, is positively biased and assists the front gate in forming a strong energy barrier [17]. This architecture eliminates the inconvenience of a single ground plane, which tends to counteract the front-gate action (Figure 2a).

*Physically-doped ungated section* (Figure 3b): The idea here is to eliminate the back-gate action. Boron implantation replaces the P* electrostatic doping, without affecting the sharpness of the device characteristics [18]. This solution is well suited to FinFETs and nanowires, although it does sacrifice some of the reconfigurability.

*Z*^3^–*FET* (Figure 3c): This Zero top-gate variant has the partitioning of the body doping controlled by the twin ground-planes [19]. The omission of the gate stack enables operation at higher voltage, without dielectric reliability issues. Furthermore, the free surface can be used for superior detection of light, biochemical species, or radiation.

### 1.4. Implementation in FD-SOI Technology

**Figure 4:**
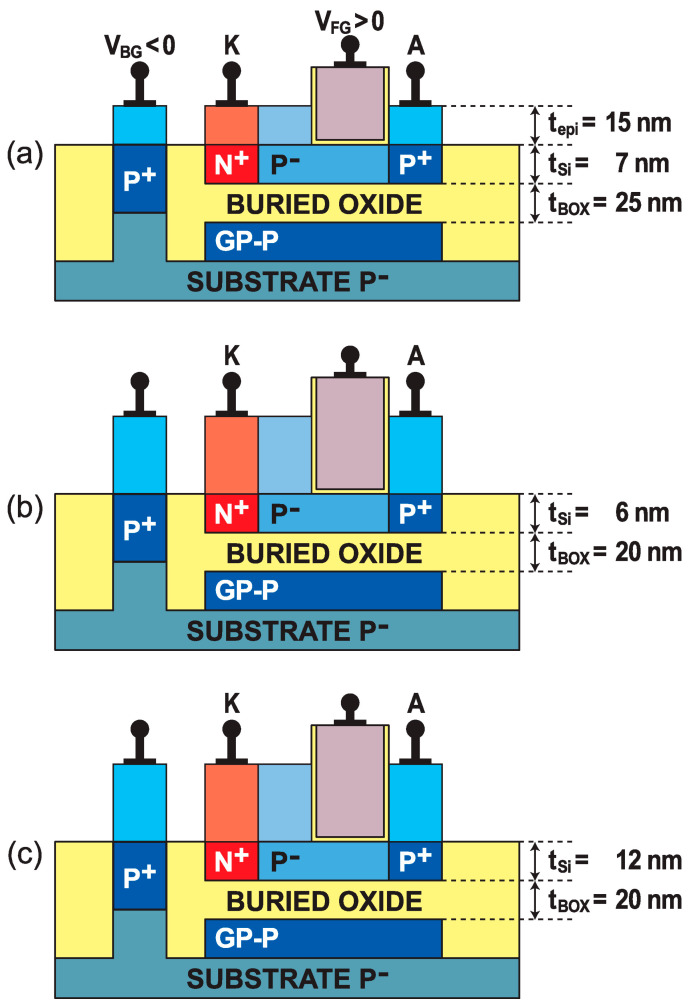
Cross-section views of Z^2^–FETs fabricated with FD-SOI technologies: (**a**) 28 nm node, (**b**) thin 14 nm node, and (**c**) thick 14 nm node.

Band modulation devices have been designed and fabricated in 14 nm [20] and 28 nm [21] FD-SOI technologies. Both advanced nodes feature promising characteristics, with a slightly different process flow. For instance, in 28 nm FDSOI, the fabrication of a Z^2^–FET structure (Figure 4a) starts by delimiting the device surface using STI isolation. A 25-nm-thick BOX separates the undoped ultrathin silicon film (t_Si_ = 7 nm with ~10^16^ cm^−3^ doping concentration) from a highly P-type doped ground plane (~10^18^ cm^−3^), which prevents the deep depletion of the substrate and acts as a back-gate. In order to access the GP and control the well potential, the BOX is locally etched. The undoped Si film of the Z^2^–FET is partially covered by a single high-k/metal front-gate with a capacitance equivalent thickness (CET) of 3.7 nm, whereas the rest of the channel is ungated. Thinner dielectrics, down to CET = 1.1 nm, have been successfully tested at lower gate voltages. The gate stack formation starts with a thermally grown oxide interface (SiO_2_), ensuring a good interface quality, clean of fixed charges. The interface SiO_2_ layer (IL) is covered by a high-k hafnium dioxide layer (HfO_2_, 18 < k < 20), improving the current drive and reducing the gate leakage. It is connected via a mid-gap metal layer (TiN or AlTiN) to a low-resistance poly-Si electrode followed by gate patterning and offset spacer formation.

After completion of the second spacer, the anode and the cathode are implanted with boron and phosphorus (10^20^ cm^−3^), respectively. An epitaxial layer (t_epi_ = 15 nm) is grown on the whole structure, to reduce series resistance. A final step, before contact formation, consists in covering the ungated region with an oxide layer (RPO), preventing silicidation.

Unlike the 28 nm process, in 14 nm FD-SOI technology, the silicon film and the BOX are reduced down to 6 nm and 20 nm, respectively (Figure 4b). The CET of the high-k/metal gate is reduced to 3.4 nm and, for low-voltage operation, to ~1 nm. A dual in situ doped (Si:CP/SiGeB) epitaxy exclusively covers the source and drain areas. Moreover, the anode and cathode contacts (sometimes called source and drain) take the form of trenches, permitting integration with a higher density of local interconnections between devices, without resorting to the first metal level. In order to study the impact of silicon film thickness, a variant with a thicker body was also fabricated (t_Si_ = 12 nm, Figure 4c).

## 2. Basic Characteristics

### 2.1. Operation in DC Mode

**Figure 5:**
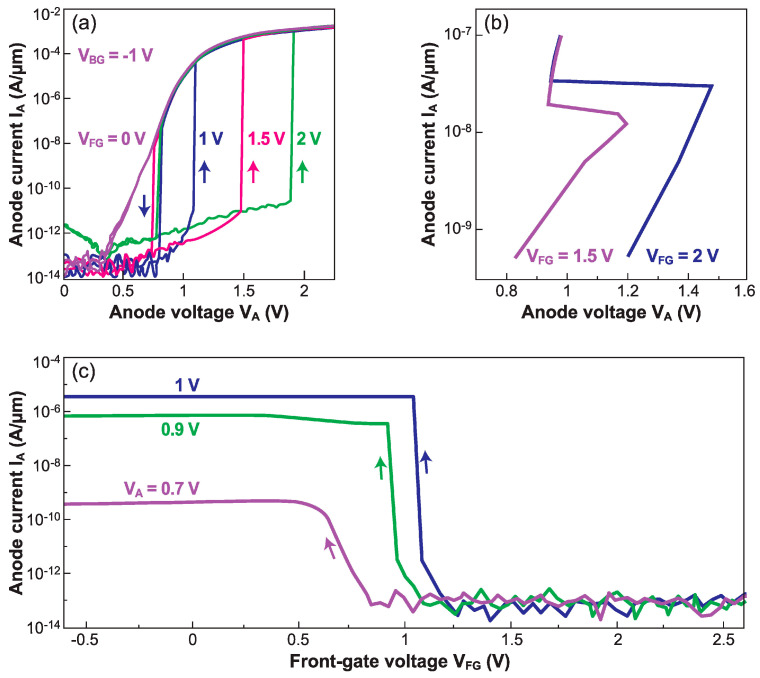
Experimental sharp-switching characteristics of various Z^2^–FETs in quasi-steady-state operation. Output I_A_–V_A_ curves for different gate voltages, measured by (**a**) scanning the anode voltage and (**b**) forcing the anode current. (**c**) Transfer I_A_–V_FG_ curves for various anode voltages V_A_.

The band-modulation effect gives rise to a sharp transition, from low to high current, in both output I_A_–V_A_ and transfer I_A_–V_FG_ characteristics. The I_ON_/I_OFF_ ratio can exceed 8 decades. Figure 5a reproduces the typical hysteresis in I_A_–V_A_ curves recorded by sweeping the anode voltage. The hysteresis results from an S-shaped characteristic that can be measured by setting the anode current with a current source (Figure 5b). For low V_A_, the barriers induced by the gates keep the Z^2^–FET in the OFF state. When V_A_ reaches a turn-on value V_ON_, the positive feedback mechanism makes the injection barriers collapse, and the current abruptly attains the normal I_ON_ value of a forward-biased PIN diode (ON state) [22]. The switching mechanism is related to the change in the minority carrier concentrations (holes in the gated N-region and electrons in the ungated P-region). In the hysteresis window, two different currents are measurable for the same value of V_A_; they can be viewed as states ‘1’ and ‘0’ in a static memory (1T-SRAM) or in a dynamic memory (1T-DRAM), as discussed in Section 5.1 and 5.2.

In Figure 5a, the hysteresis window and the turn-on voltage are enhanced by reinforcing the barrier heights, especially via V_FG,BG_. For a reverse V_A_ sweep, the device remains in the I_ON_ state until the point (V_A_ ≈ 0.7 V) where it turns off. Two equilibrium states, ‘0’ and ‘1’, are shown in Figure 5a. The hysteresis can be achieved with V_A_ and V_FG_ of around 1 V, making the Z^2^–FET a promising device for low-power circuits and embedded memory. Regarding the transfer I_A_–V_FG_ characteristics, the threshold voltage is tunable by the anode voltage and/or device dimensions.

Unlike the ultrathin FD-SOI transistors [23], the Z^2^–FET is free of supercoupling [24] and operates successfully, even in sub-10 nm thin films. Supercoupling is ineffective in Z^2^–FETs because the populations of electrons and holes are separated *laterally* (in the gated and ungated regions respectively), rather than *vertically*, as in ultrathin FD-SOI MOSFETs.

### 2.2. Transient Operation

In a transient or pulsed mode of operation, the concentrations of electrons and holes do not reach the equilibrium values and the I_A_–V_A_ characteristics differ from the behavior shown in DC operation. Consider the case where the gate is pulsed from 0 V to a positive voltage: electrons are expected to fill the gated region, but they are not readily available. Since electrons cannot be supplied promptly through the generation process, junctions, and gate leakage, the body potential is forced out of equilibrium, like in the deep-depletion regime of a MOS capacitor [2]. It follows that the energy barrier opposing the injection of holes from the anode is temporarily taller than in DC mode, hence the effective turn-on voltage V_ON_ increases. The shorter the gate pulse, the higher the V_ON_. The same mechanism raises the electron injection barrier at the cathode after pulsing the back gate.

A positive pulse on the anode is expected to attenuate the electron concentration underneath the gate. However, the change in concentration takes place in the electron recombination time scale. While carrier generation dominates the I_OFF_ region, recombination governs the turn-on and high-current regions.

**Figure 6:**
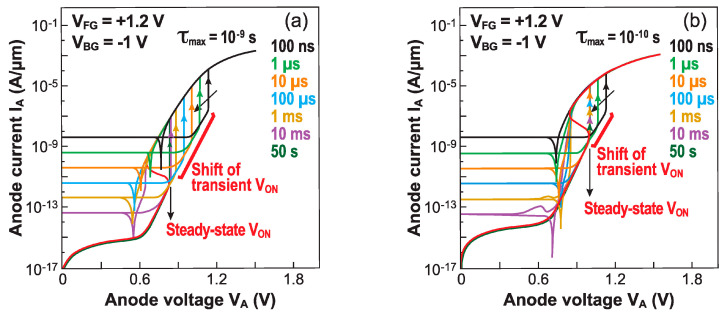
I_A_–V_A_ characteristics of Z^2^–FET for different ramp times (100 ns to 50 s) of anode voltage, from zero to 2 V. The carrier lifetime is (**a**) τ = 10^−9^ s and (**b**) 10^−10^ s. The two wells were filled with carriers by gate pulses before ramping V_A_ (adapted from [25]).

The carrier lifetime is of uppermost importance in the formation of the electrostatic barriers needed for sharp-switching. Parihar et al. demonstrated the critical role that the carrier lifetime plays in the transient operation of Z^2^–FETs [25]. TCAD simulations of their transient behavior were performed with different values of carrier lifetime. Figure 6a,b reproduce the I_A_–V_A_ characteristics for two cases (1 ns and 0.1 ns), as V_A_ is pulsed from 0 to 2 V with rise times ranging from 100 ns to 50 s (DC mode).

Faster rise times have two remarkable effects: the V_ON_ increases, widening the hysteresis window, but the leakage current in the I_OFF_ state increases as well. The latter is not an intrinsic consequence of band modulation, but rather is due to the displacement current that depends on the device capacitance and increases proportionately with the voltage ramp rate, as seen in Figure 6.

A very fast rise time does not allow the readjustment of the carrier concentration: the electrostatic barriers remain tall and block the current conduction more effectively. The change in V_ON_ depends upon the carrier lifetime, for example a longer lifetime leads to more pronounced transient effects (Figure 6a). If the lifetime is short, the carrier generation by Shockley–Read–Hall mechanisms is faster. Hence, for a 0.1 ns lifetime, the steady-state V_ON_ is achieved with a 100 μs ramp, and the shift between the transient and steady state V_ON_ values is 130 mV (Figure 6b). On the other hand, this shift in V_ON_ is 300 mV for a 1 ns lifetime and the device recovers the steady state V_ON_ with a longer ramp time of ~10 ms (Figure 6a).

## 3. Operating Principles and Models

### 3.1. Band-Modulation Mechanism

**Figure 7:**
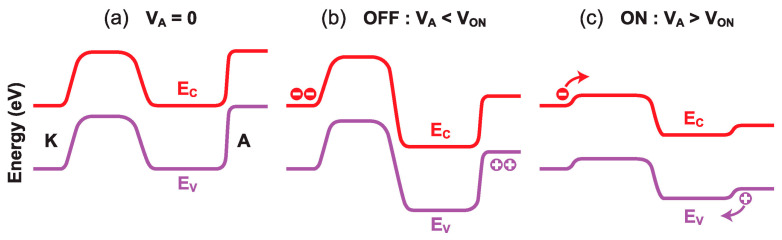
Typical profiles of the energy bands along the body as the anode voltage increases: (**a**) equilibrium at V_A_ = 0, (**b**) blocked I_OFF_ state with 0 < V_A_ < V_ON_, and (**c**) I_ON_ state with V_A_ > V_ON_. The band diagram also depends on the depth within the body, being a little different at the front and back interfaces.

The current–voltage characteristics of all band-modulation devices originate from the particular profile of the energy bands between the cathode and anode. At equilibrium (V_A_ = 0, Figure 7a), the bands undulate along the body. The height of the energy barriers at the anode and cathode is set by the front- and back-gate voltages, respectively. This is what the designation ‘band-modulation’ is meant to capture.

A positive V_A_ forward-biases both the *P*^+^–N* junction at the anode and the *N*^+^–P* junction at the cathode. It is the P*–N* electrostatic junction in-between that is reverse-biased and blocks the current flow. Any further increase in V_A_ is absorbed by this junction; the energy barriers remain tall and prevent the release of electrons and holes from their reservoirs (Figure 7b). This process continues until, eventually, the maximum voltage sustainable by the central junction is reached. At this point, a small increase in V_A_ (or a decrease in V_FG_) reduces marginally the anode energy barrier (≈V_FG_ − V_A_), enabling a few holes to fly from the anode to the cathode. Since the positive charge of holes lowers the electron injection barrier at the cathode slightly, some electrons can escape and move to the anode. Their negative charge further erodes the injection barrier for holes. A higher hole current increases the electron current and vice versa. This positive feedback process triggers the abrupt collapse of the two injection barriers and the flattening of the energy bands (Figure 7c); the device turns on suddenly for V_A_ = V_ON_, and the current increases by many orders of magnitude [26].

In the I_ON_ state, the high density of electron–hole plasma screens the gate-induced electric field and keeps the barriers flat. The Z^2^–FET behaves as a regular PIN diode, where the current increases with anode voltage, first exponentially and eventually reaching a quadratic dependence I_A_~V_A_^2^ (due to the double injection mechanism). The I_ON_ current is largely independent of the voltage applied to the gate or to the ground-plane. The device cannot be turned off until the anode voltage becomes too low (V_A_ = V_OFF_ < V_ON_) for sustaining the electron–hole plasma within the body. At that point, the energy barriers are rebuilt and the current falls sharply to zero.

The positive feedback effect is reminiscent of a thyristor. Nevertheless, the band-modulation devices are fundamentally different: (i) the doping is virtual, (ii) impact ionization does not play any role, (iii) the energy barriers are gate-controlled, and (iv) operation requires lower voltage (≈1 V) than a thyristor, leading to improved temperature stability.

In a Z^2^–FET, the front-gate voltage V_FG_ controls the hole injection barrier height, the turn-on voltage V_ON_ and the hysteresis window (V_ON_−V_OFF_). If the V_FG_ is too low and unable to build a strong energy barrier at the anode, the device acts like a PIN diode with V_ON_ ≈ 0.3 V, much smaller than the typical turn-on voltage of a silicon diode (0.8 V). Once the band modulation mechanism is restored, the turn-on voltage increases linearly with the gate voltage: V_ON_/V_FG_ = 0.8 (see Figure 5a).

**Figure 8:**
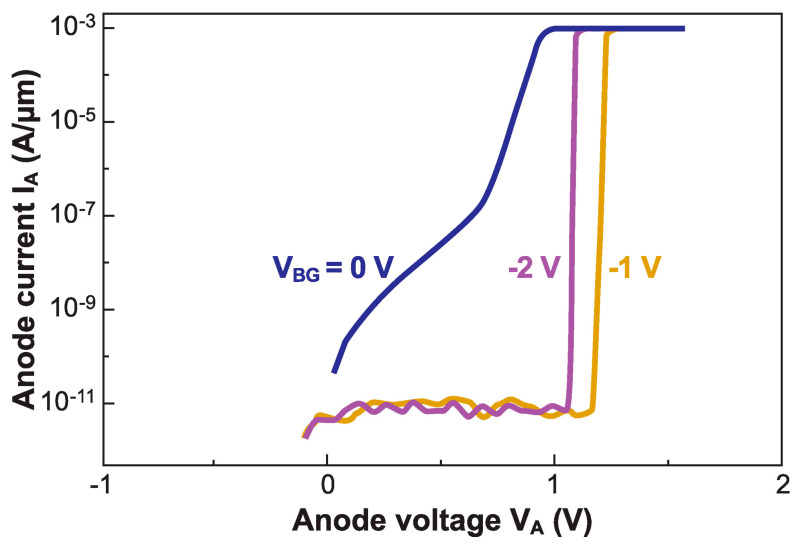
Output characteristics of a Z^2^–FET for variable voltage applied to the ground plane. V_FG_ = 1.5 V, other parameters as in Figure 4 (adapted from [27]).

As for the back gate, it controls the electron–injection barrier at the cathode, which is instrumental when the hole barrier is moderately high or the ungated region is short. Figure 8 shows a typical Z^2^–FET, which fails to operate correctly with V_BG_ = 0. A modest negative voltage (V_BG_ = −1 V) increases V_ON_, recovering the sharp switching capability and low leakage [27]. With the ground-plane extending underneath the gated region (Figure 2a), a stronger negative voltage (V_BG_ = −2 V) is actually counterproductive, as it tends to lower the hole injection barrier set by the front-gate and reduce V_ON_. This inconvenience can be eliminated with twin ground-planes (Figure 3a).

This schematic description underlines the feedback between energy barriers and carrier injection, but does not capture the full complexity of the band-modulation mechanisms in a Z^2^–FET. In the physics-based model derived by Taur et al. [28], the key consideration is the current continuity at each of the three P–N junctions: at the cathode, at the anode, and in the center of the body. The model accounts for generation-recombination (GR) and diffusion currents for electrons and holes, whereas MOS equations define the concentrations of electrons N* and holes P* (electrostatic doping) in the gated and ungated regions, respectively. The rapid variation of the quasi-Fermi levels with bias is fully captured. Five equations are sufficient to reproduce the S-shape and hysteresis of DC characteristics. The computed curve matches the experiment of Figure 5.

In the following, we focus on a compact model, suitable for circuit design.

### 3.2. Regions of Operation

**Figure 9:**
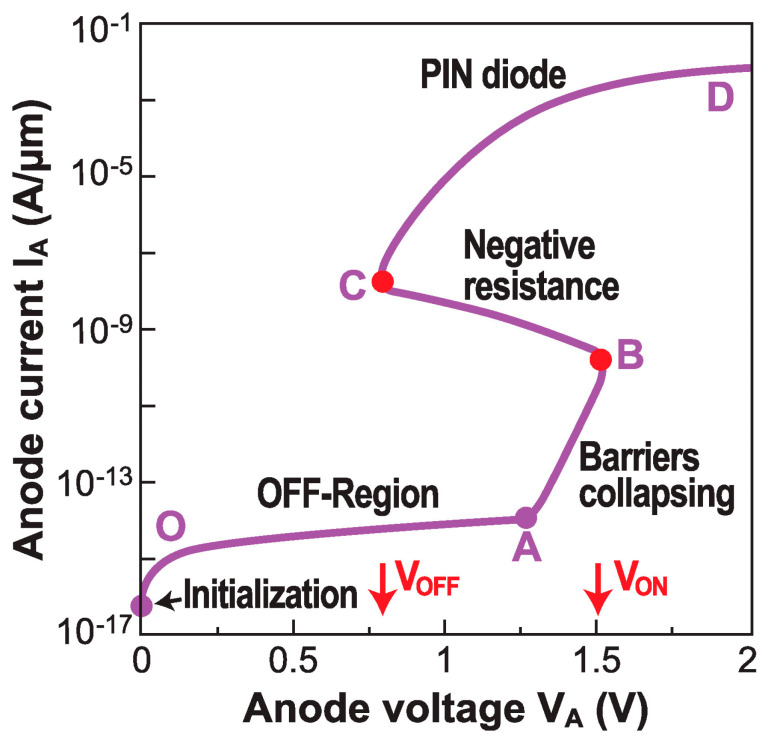
S-shaped output characteristic I_A_–V_A_ computed with Taur’s physical model [28] and Martinie’s compact model [29]. The capitals separate the five regimes in steady-state operation.

**Figure 10:**
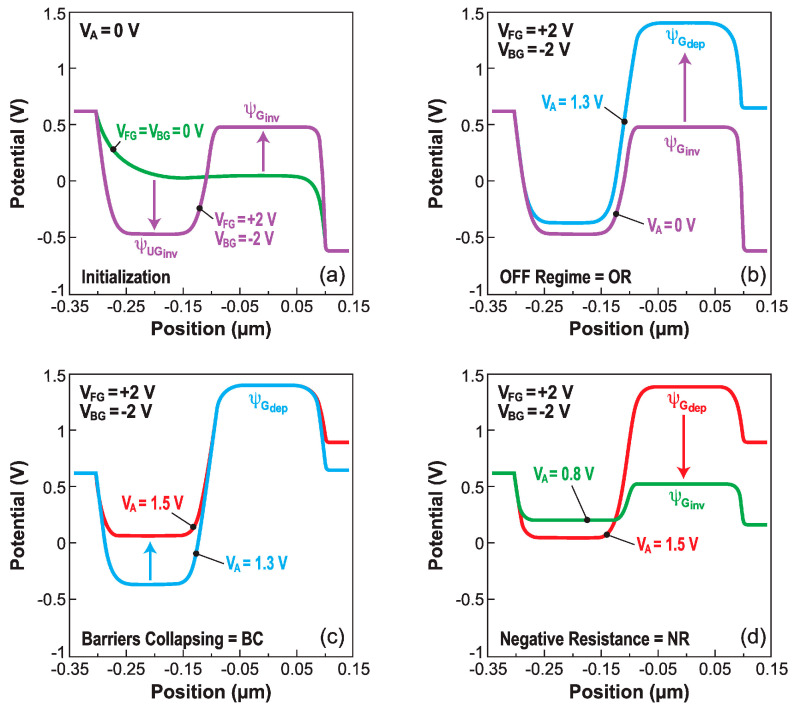
Electrostatic potential profiles from cathode (source) to anode (drain) during each DC operating regime: (**a**) initialization, (**b**) I_OFF_ region, (**c**) barrier collapse, and (**d**) negative differential resistance. TCAD simulations adapted from [29].

**Figure 11:**
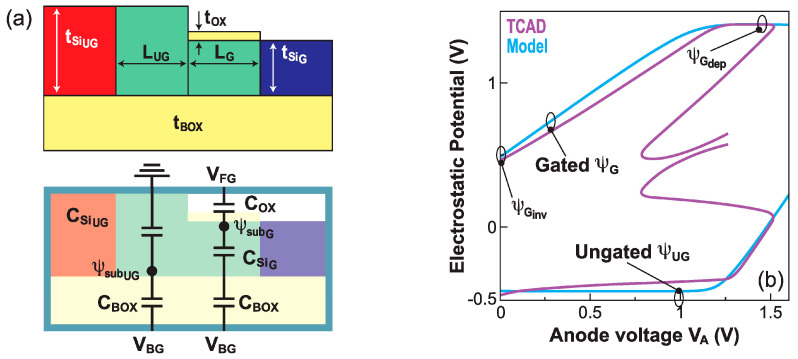
(**a**) Definition of the Z^2^–FET electrical parameters and equivalent capacitor circuit. (**b**) Electrostatic potential in gated and ungated regions vs. anode voltage in the OFF and barrier-collapsing (BC) regimes; the results derived from the model and TCAD are in good agreement (adapted from [29]).

Five regions, indicated by capital letters in Figure 9, can be identified in Z^2^–FET steady-state operation (Figure 9):

*Point O: ‘Initialization’*: The front gate and back gate are biased to produce the NPNP electrostatic structure, by transforming the PIN diode into a virtual thyristor (Figure 10a). The potential profiles are governed by the two MOS capacitors, respectively formed by (i) the gate and the overlapped body, and (ii) the back gate, the BOX, and the underlapped body (see Figure 11a).

*From O to A: ‘OFF region’*: The device is in the I_OFF_ state. The voltage drop on the anode and cathode junctions is negligible. Increasing V_A_ results in stronger reverse-biasing of the center junction, where the GR current is very low. As the *effective* gate bias (V_FG_–V_A_) decreases, the N* electron concentration tends to fall. The drain voltage increase produces a potential rise in the gated region (Figure 10b). The Z^2^–FET current is simply the leakage current of the unbiased P–N junctions.

*From A to B: ‘Barrier collapse’*: As soon as the reverse bias on the central junction reaches a maximum, the anode and cathode junctions become forward biased. Injected holes flow towards the cathode and recombine with the electrons from the cathode. The hole diffusion current dominates, which is reflected by the slope ΔV_A_/ΔI_A_ ≈ 60 mV/decade. The holes gradually accumulate in the gated region, and the electrons in the ungated region. The potential in the gated region reaches the maximum. The increase in anode voltage makes the potential in the ungated region less negative, leading to a lowering of the potential barrier between the gated and ungated regions. The central junction is less and less reverse-biased, until it eventually turns on at point *B*, corresponding to the sharp switch that defines V_ON_, as V_A_ is swept upward (Figure 10c).

*From B to C: ‘Negative differential resistance’*: The anode voltage shared by the three forward-biased junctions is unnecessarily large. The negative-resistance region means that the current increases even if V_A_ is reduced. The potential in the gated region goes from MOS depletion to inversion value. The end of this regime denotes the sharp switch under reverse bias scan V_OFF_ (Figure 10d).

*From C to D: ‘PIN diode*’: The energy barriers are demolished, and the device operates as a forward-biased PIN diode in double-injection mode. The region of very high current is affected by the series resistance, which limits the effective voltage drop on the diode.

This short description highlights that the Z^2^–FET’s behavior results from the competition between the MOS transport, driven by minority carriers, and the PIN diode with ambipolar transport. The sharp switching from I_OFF_ to I_ON_ and vice versa reflects the sudden shift from a MOS mode to PIN mode of operation.

### 3.3. Regional Model

With the Z^2^–FET being a partially gated PIN diode, its I_A_–V_A_ characteristic can be formulated using the standard PIN diode current model, given by [30]:

(1) $$I_{A}=I_{\mathrm{Dif}}\left(e^{\frac{V_{A}-R_{\mathrm{PIN}}I_{A}}{N\beta }}-1\right)+I_{\mathrm{Rec}}{\left(e^{\frac{V_{A}-R_{\mathrm{PIN}}I_{A}}{N\beta }}-1\right)}^{2}$$

where I_Dif_ and I_Rec_ respectively stand for diffusion and recombination currents, R_PIN_ represents the intrinsic body resistance, β = 𝑘𝑇/𝑞, and N is the diode ideality factor dependent on the operation regime (see [29] for details).

In the I_OFF_ regime, the applied voltage V_A_ is small, so the square term in Equation (1) can be neglected. As the drain current in this regime is insignificant, the term R_PIN_I_D_ can also be ignored. The anode current in the I_OFF_ region reduces to:

(2) $$I_{A_{\mathrm{OFF}}}=I_{{\mathrm{Dif}}_{\mathrm{OFF}}}\left(e^{\frac{V_{A}}{N\beta }}-1\right)$$

where I_DifOFF_ is a fitting parameter related to the diffusion current.

In the ‘barrier collapse’ (BC) regime, V_A_ cannot be neglected, but the drain current is low enough and the term R_PIN_I_D_ is still negligible. The first term of Equation (1) can be dropped, leading to a well-defined expression for the current I_A-BC_:

(3) $$I_{A_{\mathrm{BC}}}=I_{{\mathrm{Rec}}_{\mathrm{BC}}}\left(e^{\frac{V_{A}}{N\beta }}-1\right)2$$

where I_RecBC_ is a fitting parameter connected to the recombination current and N ≈ 2.

The ‘negative differential resistance’ regime is too complex for a physics-based model. However, noting the linear variation of the anode current logarithm in Figure 9, we can construct an empirical equation for I_A-NEG_:

(4) $$I_{A_{\mathrm{NEG}}}=\frac{I_{\mathrm{ON}}-I_{\mathrm{OFF}}}{V_{\mathrm{ON}}-V_{\mathrm{OFF}}}V_{A}+\frac{V_{\mathrm{ON}}I_{\mathrm{OFF}}-V_{\mathrm{OFF}}I_{\mathrm{ON}}}{V_{\mathrm{ON}}-V_{\mathrm{OFF}}}$$

where V_ON_ and V_OFF_ are the switching voltages under forward and reverse bias sweeps, while I_ON_ and I_OFF_ are the corresponding drain currents, just before the switch.

The keys aspect of the regional model is the calculation of the boundary conditions between each regime, namely the switching voltages V_ON_ and V_OFF_ [29].

### 3.4. Switching Voltages

Figure 10 shows the large variation of the electrostatic potential from depletion to inversion under the gate. The potential in the gated region Ψ_G_ and in the ungated body Ψ_UG_ can be formulated via an MOS-centric description.

In depletion mode, the equivalent capacitor dividers in Figure 11a yield the corresponding potentials Ψ_Gdep_ and Ψ_UGdep_:

(5) $$\psi_{G_{\mathrm{dep}}}=\frac{C_{\mathrm{BOX}}\cdot C_{\mathrm{Si}}\cdot \left(V_{\mathrm{BG}}-V_{\mathrm{FG}}\right)}{C_{\mathrm{OX}}\cdot C_{\mathrm{Si}}+C_{\mathrm{BOX}}\cdot C_{\mathrm{Si}}+C_{\mathrm{OX}}\cdot C_{\mathrm{BOX}}}+V_{\mathrm{FG}}$$

(6) $$\psi_{{\mathrm{UG}}_{\mathrm{dep}}}=\left(\frac{C_{\mathrm{BOX}}}{C_{\mathrm{BOX}}+C_{{\mathrm{Si}}_{\mathrm{UG}}}}\right)V_{\mathrm{BG}}$$

The inversion mode is mathematically more complicated. The gated region is a classical FD-SOI MOS structure, where the explicit solution from the Leti–UTSOI model applies [31]. The ungated region behaves like a bulk device, with limited substrate depth and thick gate oxide (the BOX), mainly driven by the back-gate bias. The Poisson integration is simplified by considering only the back channel in the ungated region. The explicit solution proposed in [29] reads:

(7) $$\psi_{\mathrm{UG}}=\psi_{{\mathrm{UG}}_{\mathrm{dep}}}-2\beta \cdot \mathrm{LW}\left(\frac{\sqrt{q\cdot \varepsilon_{\mathrm{Si}}\cdot \mathrm{ni}}}{\sqrt{2\beta }\cdot C_{\mathrm{BOX}}}\cdot e^{\frac{\psi_{{\mathrm{UG}}_{\mathrm{dep}}}-\mathrm{Vc}}{2\beta }}\right)$$

where the analytical formulation of the Lambert function is

$$\mathrm{LW}\left(x\right)=\ln\left(1+x\right)\left[1-\frac{\ln\left(1+\ln\left(1+x\right)\right)}{2+\ln\left(1+x\right)}\right]$$

The quasi-Fermi level potential V_C_ is deduced from TCAD simulations. Figure 11b illustrates the complex variation of the electrostatic potentials Ψ_G_ and Ψ_UG_ with anode voltage. The validity of the proposed model is confirmed by a comparison with the TCAD results.

**Figure 12:**
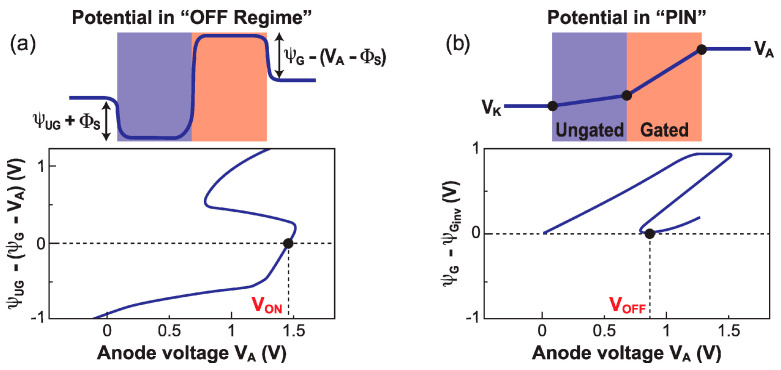
Schematic view and potential variation vs. anode voltage in (**a**) the I_OFF_ regime and (**b**) PIN regime, showing the position of the switching voltages V_ON_ and V_OFF_ (adapted from [29]).

The turn-on voltage V_ON_ corresponds to the sharp switching point in the increasing V_A_ sweep, that is the boundary voltage between the barrier collapse and negative differential resistance regimes (point B in Figure 9). The potential variation Ψ_UG_ − (Ψ_G_ − V_A_) monitors the difference between the pseudo build-in voltages in the junctions formed by the cathode and the ungated body (Ψ_UG_) and by the anode and the gated region (Ψ_G_ − V_A_). Figure 12a shows that the switch point occurs when this differences is close to 0, wherever this happens within the silicon film. An iterative calculation yields a first order approximation of V_ON_:

(8) $$V_{\mathrm{ON}}=\psi_{G_{\mathrm{dep}}}-0.5\psi_{\mathrm{UG}}\left(\psi_{G_{\mathrm{dep}}}\right)-0.5\psi_{\mathrm{UG}}\left(\psi_{G_{\mathrm{dep}}}-0.5\psi_{\mathrm{UG}}\left(\psi_{G_{\mathrm{dep}}}\right)\right)$$

where Ψ_Gdep_ is the potential in the depleted gated region (Equation (5)) and Ψ_UG_(V_A_) is the potential in the ungated region as a function of anode voltage; an analytical expression of Ψ_UG_(V_A_) is derived in [29].

Conversely, the turn-off voltage V_OFF_ is the boundary voltage between the negative differential resistance and PIN regimes (point C in Figure 9). The device turns off when V_A_ is sufficiently low to allow the gate to regain control of the gated region, as shown in Figure 12b. The lower limit of the PIN regime occurs when the potential difference Ψ_G_ − Ψ_Ginv_ reaches zero. To obtain an analytical expression for Ψ_G_, Ohm’s law can be applied to the gated and ungated segments, finally leading to V_OFF_:

(9) $$V_{\mathrm{OFF}}=\frac{2t_{{\mathrm{si}}_{\mathrm{UG}}}+2t_{{\mathrm{si}}_{G}}}{t_{{\mathrm{Si}}_{\mathrm{UG}}}+2t_{{\mathrm{Si}}_{G}}}\psi_{\mathrm{Ginv}}$$

V_ON_ and V_OFF_ are primarily dependent on the potential in the gated region. The excursion range of V_ON_ is quite wide, because it depends, in the first order, on the depleted surface potential that exhibits a large variation with V_FG_. On the other hand, the V_OFF_ excursion range is modest, since it depends on the *inverted* surface potential that varies only weakly with V_FG_.

### 3.5. Compact Model V-I Formulation

The main challenge for compact modeling is a unified description of the steady-state hysteresis. This means that the same equations must capture all biasing schemes. This cannot be achieved by the current-voltage I_A_(V_A_) formulation, as proposed in the previous regional model. Instead, the reciprocal voltage-current V_A_(I_A_) characteristics can be analytically constructed from the following set of equations:

(10) $$V_{A_{\mathrm{OFF}}}=N_{\mathrm{OFF}}\cdot \beta \cdot \ln\left(1+\frac{I_{A}}{I_{{\mathrm{Dif}}_{\mathrm{OFF}}}}\right)$$

(11) $$V_{A_{\mathrm{BC}}}=N_{\mathrm{BC}}\cdot \beta \cdot \ln\left(1+\sqrt{\frac{I_{A}}{I_{{\mathrm{Rec}}_{\mathrm{BC}}}}}\right)+N_{\mathrm{BC}}\left(\psi_{G}{}_{\mathrm{dep}}-\psi_{G}{}_{\mathrm{inv}}\right)$$

(12) $$V_{A_{\mathrm{NEG}}}=\frac{V_{\mathrm{ON}}-V_{\mathrm{OFF}}}{I_{\mathrm{ON}}-I_{\mathrm{OFF}}}I_{A}+\frac{V_{\mathrm{ON}}I_{\mathrm{OFF}}-V_{\mathrm{OFF}}I_{\mathrm{ON}}}{I_{\mathrm{ON}}-I_{\mathrm{OFF}}}$$

(13) $$V_{A_{\mathrm{PIN}}}=N_{\mathrm{PIN}}\cdot \beta \cdot \ln\left(\frac{2\cdot I_{{\mathrm{Rec}}_{\mathrm{PIN}}}-{\;I}_{{\mathrm{Dif}}_{\mathrm{PIN}}}+\sqrt{I_{\mathrm{Dif}}^{2}+4I_{A}I_{{\mathrm{Rec}}_{\mathrm{PIN}}}}}{I_{{\mathrm{Rec}}_{\mathrm{PIN}}\;}}\right)+R_{\mathrm{PIN}}I_{A}$$

**Figure 13:**
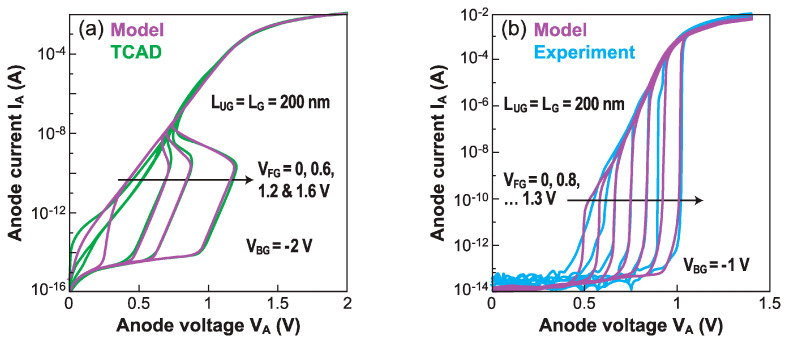
(**a**) Current-driven Z^2^–FET characteristics predicted by the compact model and by TCAD simulations. (**b**) Voltage-driven characteristics obtained from model and measurements (adapted from [29]).

Each of the above four equations is valid in a well-defined region, designated by its subscript: OFF for off-current regime, BC for barrier collapse regime, NEG for negative resistance regime, and PIN for the conductive diode regime. Thanks to the analytical expressions for the boundary conditions between the conductive and blocked regimes (i.e., V_ON_ and V_OFF_), Equations (10)–(13) are linked in a common formulation to ensure continuity (mandatory for implementation in Verilog-A). Figure 13 shows a convincing agreement of the model with, both, TCAD results (a) and experimental data (b).

## 4. Sharp-Switching Performance

**Figure 14:**
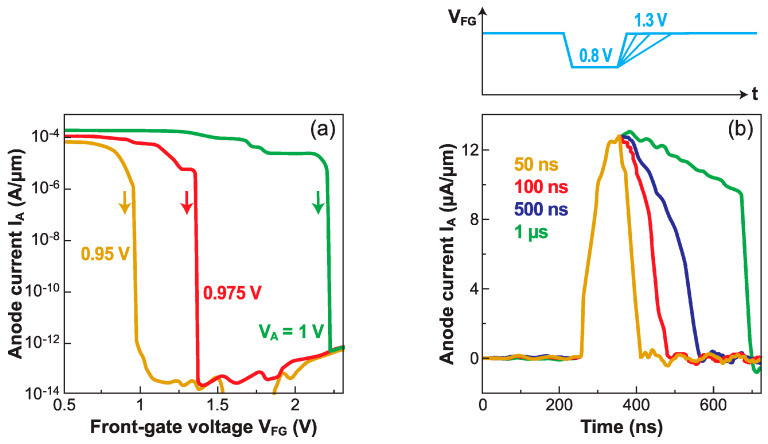
(**a**) Turn-off transfer characteristics in DC mode. (**b**) Current switch vs. time in response to gate pulses with variable rise time shown above (V_FG_ from 1.3 V to 0.8 V and back): V_A_ = 0.8 V, t_si_ = 7 nm, t_BOX_ = 25 nm, L_G_ = L_UG_ = 500 nm, adapted from [32].

The steady-state transfer characteristics I_A_(V_FG_) in Figure 5c demonstrate exceptionally sharp turn-on capability. The switching to I_OFF_ is also abrupt, but more challenging because the energy barriers need to be reconstructed. The gate voltage required to turn off the Z^2^–FET is markedly higher than to turn it on. This hysteresis becomes larger as the anode voltage increases. Figure 14a shows that a small increase of 50 mV in V_A_ (from 0.95 V to 1 V) requests a more than 1 V increase in V_FG_, to abruptly suppress the current. A too high anode voltage renders the gate unable to turn off the device. These problems can be avoided by using fast pulses on the gate [32].

Figure 14b shows the current variation in the time domain in response to gate pulses of 0.5 V amplitude. The device turns sharply from an I_OFF_ to I_ON_ state as V_FG_ is pulsed down. Then, pulsing the gate voltage up, turns the device off. A fast gate pulse, together with the absence of hysteresis, is essential for logic circuits. It is worth noting that during the rise time of the gate signal, the electron concentration cannot be adjusted promptly enough, and the hole barrier is temporarily taller than in DC mode. This explains why the gate voltage needed to block the device is much smaller in pulsed mode than in DC mode. As a result, a gate pulse of 0.5 V is only effective in switching the device back and forth.

Systematic measurements reveal the importance of the pulse time constants. It is the collapse of the hole injection barrier that makes the device turn on. The discharge current that sweeps away the electrons stored under the gate contributes to the feedback mechanism that suddenly collapses the barrier. This discharge current is obviously amplified for very short fall times of the pulse.

The device turn-off is less spontaneous and critically dependent on the rise time of the pulse. In Figure 14b, a relatively long pulse (>1 µs) is inefficient, as it tends to keep the device locked in conductive mode with high leakage-like current. Instead, a very short pulse (<50 ns) produces a perfect spike in current. Very fast rise times succeed in restoring tall energy barriers, before the anode and cathode can supply fresh holes and electrons. This means that switching the device off requires a pulse rise time shorter than the transit time of carriers between the terminals. Simulations predict successful operation in the pico-second range.

**Figure 15:**
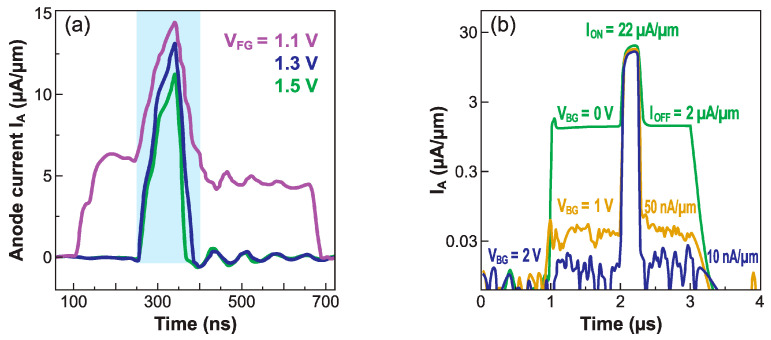
Current switch in response to a gate pulse showing the impact of (**a**) front-gate voltage (for V_A_ = 0.8 V) and (**b**) ground-plane voltage V_BG_ (V_A_ = 1.1 V, V_FG_ = 0.5 V, adapted from [32]). The gate pulse has a 0.5 V amplitude and varies, as in Figure 14b, between the indicated V_FG_ value and a lower level.

The effectiveness of the switch depends on the balance between the anode and front-gate voltages. Figure 15a shows a Z^2^–FET biased with 0.8 V on the anode. Ideal switching properties, without leakage, are measured when the upper level of the gate pulse is higher than 1.3 V. A lower gate voltage (V_FG_ = 1.1 V) is insufficient to build strong energy barriers that would block the device; the minimum current in the OFF state is no longer zero, leading to unacceptable leakage. Increasing the anode voltage to ensure a higher I_ON_ (increasing V_A_ by 0.2 V roughly doubles the current drive) makes the barrier reconstruction more difficult, leading to rising leakage currents. The solution is to concomitantly increase the gate pulse.

The fine-tuning of the ground-plane voltage offers yet another solution for the suppression of the leakage current. However, a too negative V_BG_ counteracts the effort of the front-gate to set a strong hole-injection barrier at the anode, whereas a too positive V_BG_ hampers the creation of an electron barrier at the cathode. Once again, the dual ground-plane variant (Figure 3a) shows improved performance [32]. It is the ground-plane located beneath the gated region that makes the difference. When V_BG_ = 0 the leakage is high, but it decreases by orders of magnitude for V_BG_ = +2 V (Figure 15b). This ground-plane can guarantee a strong barrier at the anode, even though the front-gate voltage is kept small (V_FG_ = 0.5 V), suitable for low-voltage applications. Note that the ground-plane biasing does not impact the I_ON_ current. The I_ON_/I_OFF_ current ratio can exceed six orders of magnitude. The proper selection of anode, gate, and ground-plane voltages is even more crucial in short-body devices, where the energy barriers are narrower and, thus, more transparent. While I_ON_ current is improved, the I_OFF_ leakage current tends to increase more quickly unless the gate voltages are increased.

In summary, the Z^2^–FET stands as a surprisingly solid contender in the arena of sharp-switching devices. Its main asset is its performance at very high frequencies: (i) sharp turn-off enabled by higher energy barriers in the pulsed mode, (ii) easy turn-on thanks to the gate discharge current, and (iii) short I_ON_ state duration, to save energy. A further aspect appealing for low-power applications is the devices capability to switch on and off at gate and drain voltages in the sub-1V range.

## 5. Alternative Applications of Sharp Switching

### 5.1. Static Memory Cell: Operation as Capacitorless SRAM

**Figure 16:**
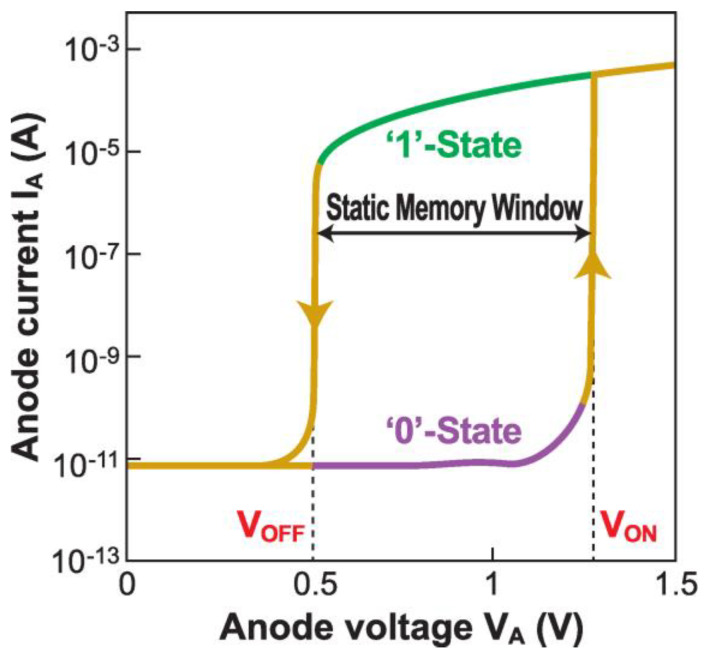
N-type Z^2^–FET DC curves showing the static memory operation. The anode current needed to hold the ‘1’-state limits the application of Z^2^–FET-based single-transistor SRAM to low-density chips.

The DC hysteresis observed when sweeping the Z^2^–FET anode voltage back and forth while monitoring the current suggests the possibility of using the Z^2^–FET as a single-transistor static RAM cell [26]. The static memory window, i.e., the valid anode voltages to operate the cell as an SRAM, is defined as the range between the DC current switching points V_ON_ and V_OFF_, see Figure 16. The high-current ‘1’-state is set by applying V_A_ > V_ON_, to overcome the injection barriers and forward-bias the cell. In contrast, the low-current ‘0’-state is programmed with a low anode voltage V_A_ < V_OFF_; the barriers are rebuilt and block the current flow. In either case, after setting the ‘1’- or ‘0’-state, the anode bias must be maintained within the limits of the memory window, V_OFF_ < V_A_ < V_ON_, to avoid flipping the memory state.

The large hysteresis, with excellent stability during memory cycling, the ease of operation, with only the anode contact as a control terminal, and the unrivalled current margin between states would seem to make the Z^2^–FET cell a promising candidate for SRAM applications. The critical drawback is related to the power consumption in the ‘1’-state. Unlike the ‘0’-state, where the current is practically zero, the ‘1’-state is associated with a relatively high I_ON_ since the anode bias cannot be reduced below V_OFF_. Unless the Z^2^–FET is somehow combined with a current blocking device, the power consumption would be unsustainable in billion-cell SRAM chips.

### 5.2. Dynamic Memory Cell: Operation as Capacitorless DRAM

**Figure 17:**
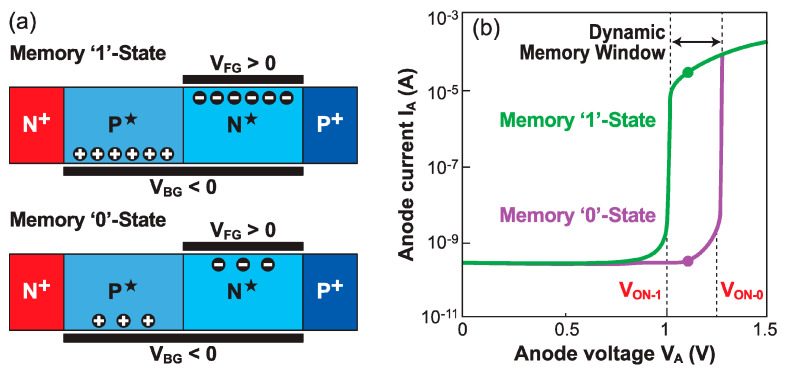
Operation of N-type Z^2^–FET DRAM. (**a**) Carrier populations in ‘1’ and ‘0’ memory states. (**b**) Output characteristics in ‘1’ and ‘0’ states, showing the measured currents (adapted from [34]).

The Z^2^–FET stands out as a competitive capacitor-less DRAM cell [13], especially for embedded applications, due to its full compatibility with standard FD-SOI CMOS process flow and low-voltage designs [33]. Its dynamic memory operation relies on transient modulation of the onset voltage V_ON_ [34]. It is necessary to form the horizontal doping profile of the virtual NPNP Shockley diode (i.e., floating-body thyristor), by biasing the gate terminals [11]. The turn-on voltage V_ON_ is modified by altering the carrier population within the body of the device. Normally, a high carrier density under the top gate, as in the DC regime, lowers the device turn-on voltage V_ON-1_ as the height of the barrier is reduced. This is the standard memory ‘1’-state of the Z^2^–FET. However, a short pulse on the front gate cannot quickly populate the body underneath, leading to a deep-depletion regime that yields an increased potential barrier, corresponding to a higher triggering voltage V_ON-0_ [35]. This would be the ‘0’-state. The difference (V_ON-0_–V_ON-1_) defines the dynamic memory window, with a typical value of 200–300 mV (Figure 6). The Z^2^–FET memory cell can be read out with an anode voltage pulse in the V_ON-0_ < V_A_ < V_ON-1_ range: the current is high in the ‘1’-state (V_A_ > V_ON-1_) and negligible in the ‘0’-state (V_A_ < V_ON-0_) [34]. Figure 17a depicts the Z^2^–FET dynamic memory for an *n*-type device (top gate located beside the *P*^+^ anode terminal), with virtual body doping induced by the top (V_FG_) and bottom (V_BG_) gates.

In any DRAM cell, four main operations are performed: read, write, erase, and hold. The memory readout process (R) aims at discriminating the cell states based on the current level. The readout anode voltage V_A_ is set inside the dynamic memory window (see Figure 17b). In the ‘1’-state, the Z^2^–FET essentially behaves as a forward-biased PIN diode, with a large output current. Conversely, in the ‘0’-state, the central PN junction is reverse-biased and passes a very low leakage current. Hence extremely high current ratios I_1_/I_0_ can be obtained, enabling easy memory state discrimination. It is worth noting that, as in most capacitorless alternatives (MSDRAM, A2RAM, ZRAM, … [36,37]), the readout process is based on current rather than voltage sensing.

The holding phase (H) is expected to preserve the programmed memory states for as long as possible. A natural trade-off arises as a consequence of the dual goal of this operation; maintaining a high carrier concentration in the ‘1’-state and a low carrier concentration in the ‘0’-state. To save energy, the anode is grounded while the gates are biased to ensure the corresponding memory-state barrier conditions.

Finally, the mechanisms to produce suitable carrier populations underneath the gate are known as write (W) and erase (E) operations. The carrier storage needed to program ‘1’ is achieved by grounding the gate, thereby lowering the injection barrier, and then forward-biasing the cell with an anode pulse to allow carrier flow. The sudden building of the barrier with a positive top-gate bias pulse captures the flowing electrons under the gate. On the other hand, the carrier evacuation for writing the ‘0’-state is achieved through capacitive coupling, by grounding the top gate without any anode pulse; the carriers under the front gate are, thus, expelled [13].

**Figure 18:**
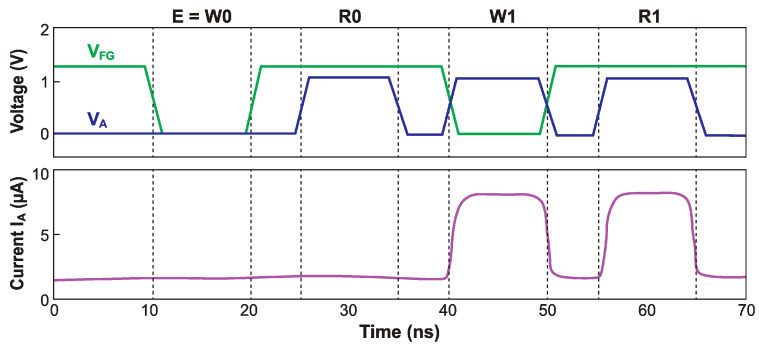
Z^2^–FET dynamic memory operation consisting of a E-R-W-R sequence. The hold operation (not indicated) is performed by default after any other operation. The cell drives current only for the ‘1’-state programming (W1) and readout (R1).

Figure 18 shows an example of voltage pattern with all four operations and the anode current readout as a function of time.

With a ‘0’-state programmed, the deeply depleted body tends to get repopulated with carriers, due to thermal generation and parasitic current injection. By contrast, in the ‘1’-state the excess carrier concentration may decrease with time, due to recombination and current leakage. These two processes make the turn-on voltage shift with time and the dynamic memory window becomes narrower. When the turn-on voltages for logic ‘1’ and ‘0’ merge (V_ON-0_ = V_ON-1_), the memory effect vanishes [34]. Therefore, the carrier charge under the front gate needs to be periodically refreshed, lest the memory state is lost. In particular, the unwanted population of electrons that tends to accumulate under the gate and eventually corrupt state ‘0’ can be eliminated by grounding the front gate.

The maximum elapsed time in which it is still possible to recover the memory state without losing the information is known as the retention time. Frequent readout of the ‘0’-state improves retention, because the parasitic electrons are swept away by the anode pulse.

The performance of the Z^2^–FET as a 1T-DRAM cell can be briefly summarized, as follows:

**Speed**: The Z^2^–FET DRAM performs best when the program and readout pulses are short. The power dissipation is obviously reduced by limiting the time I_ON_ flows (when reading out the ‘1’-state). Measurements show that with 320 ns anode pulses, the energy saving is two orders of magnitude compared with a 120 µs pulse [22]. Ultra-fast operation has been demonstrated through TCAD simulations, with access times below 1 ns. The performance is enhanced for two reasons: (i) shorter anode pulses enable lower readout voltages, and (ii) shorter top-gate pulses enhance the barrier height. Besides, slow cell access times may result in the loss of the sharp-switching capability.

**Retention time**: Both memory states benefit from repeated readout operation, which has a regenerative effect. Adequate control of the carrier concentration during the ‘0’-state hold operation is essential. After careful optimization, retention times over 500 ms have been demonstrated [38]. Trap-assisted tunneling [39] and especially carrier generation through Shockley–Read–Hall (SRH) process at the anode-body junction [40] seem to be the main limiting factors to extending the retention time. Some techniques have been explored to improve it, for example the half-ground-plane Z^2^–FET [41] or the double ground-plane (Figure 3a).

**Energy consumption**: The Z^2^–FET DRAM current is only significant when writing or reading the ‘1’-state, see Figure 18. A high ‘1’-state read current is attractive for state discrimination. Therefore, optimization efforts have focused on the energy reduction for ‘1’-state programming. It has been demonstrated that it is possible to write the cell with low anode-voltage (V_A_ ≈ 0.4 V), greatly reducing the current and practically suppressing the programming energy [42]. Energy and power consumption now occur exclusively during the readout of the ‘1’-state, with less than 1pJ/bit per operation at 1 V and an even lower standby energy. Moreover, the ‘1’-state readout process regenerates the ‘1’-state condition, relaxing the need for periodic refreshing. The use of alternative materials with a narrow bandgap, such as germanium, is an attractive avenue towards reducing the operating voltage and, hence, the power consumption of Z^2^–FET DRAM cells. This has been demonstrated through numerical simulations [43].

**Temperature dependence**: Tests under elevated temperature conditions have proven that successful operation is possible, although some minor modifications may be required. The trigger voltage V_ON_ shifts slightly to lower voltages as a consequence of the increased carrier energy, which eases the injection over the energy barriers [33,38]. This enables the use of Z^2^–FET cells with lower biasing conditions and power consumption. As a drawback, increasing the temperature augments the SRH generation–recombination, reducing the retention time [39].

**Reliability, noise, and variability**: Metrics such as the DC V_ON_ and I_ON_, the dynamic memory window, and the retention time for the Z^2^–FET were experimentally studied at high temperature (85 °C) for different cell geometries and accelerated stress conditions. The V_ON_ voltage shifts up to 50 mV after long stress periods. It was also found that I_ON_ is affected by bias-temperature instabilities (BTI) and random telegraph noise (RTN) fluctuations [44,45]. Interface and oxide trapping effects are thought to be responsible. Experimental time-dependent dielectric breakdown (TDDB) analysis demonstrated lower breakdown variability for wider cells, but a shorter time to breakdown, indicating a trade-off between reliability and variability [44]. A series of 3D TCAD simulations were conducted to analyze the variability impact of fast states at each interface. Interface states play a fundamental role in the device sharp-switching behavior, through the carrier lifetime value [25,46]. An excessive density of interface defects at the top of the ungated region degrades the capacitorless operation, increasing the V_ON_ and the variability. This effect was found to be more critical as the cell was scaled down [47]. Nevertheless, a proper design strategy could alleviate the impact of high interface state densities in short cells and recover the sharp-switching capability at the expense of larger biasing voltages.

**Scaling**: TCAD simulations predict that the gate of the device can be scaled down to 30 nm [26] with the aid of GP-bias tuning [48]. Unfortunately, to preserve good electrostatic control, the ungated region cannot be downscaled as aggressively. Nonetheless, the Z^2^–FET cell is comparable in terms of area per bit with advanced embedded DRAM that inherently includes a storage capacitor [48]. The geometric scaling enables enhanced oxide reliability, due to the dielectric surface reduction [44] but, as a drawback, it may degrade the retention time and the variability [47], demanding additional bias tuning to overcome this issue.

**Matrix operation**: The operation of the Z^2^–FET cell in an array configuration was first studied through simulations [49] and later characterized experimentally in a two-by-two cell matrix, with and without, a pass selector transistor [50]. Single-cell and word-length operations were successfully demonstrated at low [51] and high temperature [52], with promising immunity to bitline or wordline disturbances and an easy-to-scale design. A prototype 1 Mbit memory chip, fabricated with FD-SOI technology, proved fully functional, with reliable performance.

### 5.3. Protection against Electrostatic Discharge

**Figure 19:**
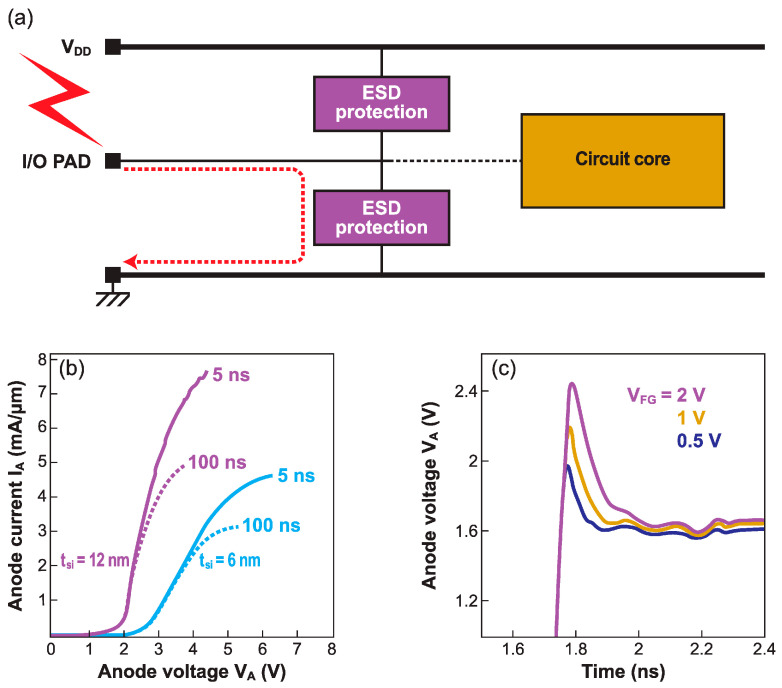
(**a**) Schematic ESD protection with a Z^2^–FET. (**b**) Output characteristics from TLP tests with long and short current pulses. (**c**) Anode voltage response in the very first nanoseconds after ESD strike showing the voltage overshoot (1 mA/µm current pulse with 55 ps rise time, adapted from [27,53]).

Hazardous electrostatic discharge (ESD) on chip pads should be diverted by a protection device before they reach and damage the core circuitry (Figure 19a). An optimum protection is expected to respond quickly, by switching from a sleep mode (OFF) to a high current-drive I_ON_ mode. Devices with an S-shaped current–voltage characteristic, operating in a certain voltage window, defined by the technology node and chip biasing, are of particular interest. This is exactly what band-modulation devices are able to do.

A fundamental advantage is the tunability of the turn-on voltage via both the front-gate and back-gate voltages. The characteristics of the Z^2^–FET in Figure 5a,b are perfectly suited for ESD protection. Whatever maximum voltage V_BD_ can be tolerated by the chip before breakdown and fatal damage, the trigger voltage can be adjusted to V_ON_ = 0.9 V_BD_.

ESD tests are conducted with transmission line pulse (TLP) methodology, which records the voltage drop produced by a brief pulse of anode current. Reconstructed I_A_(V_A_) curves are shown in Figure 19b. A Z^2^–FET with a thicker body (12 nm) can handle a current of 8 mA/µm [27]. Relatively long (100 ns) ESD discharges generate considerable self-heating in FD-SOI films, which lowers the maximum current. However, the advantage of ultrathin films over bulk silicon comes from a significantly faster response (120 ps) and a reduced overshoot voltage [53]. Figure 19c shows the waveform of the anode voltage just after an ESD event. The temporary voltage overshoot, where the device might be vulnerable, is minimized to 0.8 V.

### 5.4. Photo-Detection

The operation of the Z^2^–FET as a photodetector takes advantage of the same nonequilibrium mechanism used in 1T-DRAM. The generic principle is to bias the device such that it stays in blocked mode (‘0’-state) in the dark and turns on (‘1’-state) during illumination. Light is detected by the abrupt switch from low to high anode current. The turn-on voltage under illumination is the sensing parameter [54,55].

In order to enhance the light absorption efficiency, rather thick top silicon film (t_si_ = 200 nm) is used in this simulated device. The gated and ungated regions are 0.5 µm and 1.5 µm long and the BOX is 500 nm thick.

**Figure 20:**
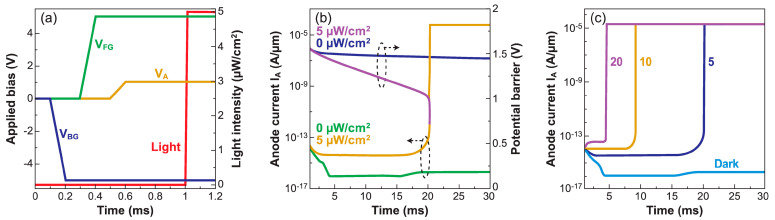
(**a**) Bias and light waveforms in a Z^2^–FET photo-detector. (**b**) Time-dependent evolution of the potential barrier at anode and Z^2^–FET current with and without exposure to light at *t* = 1 ms. (**c**) Anode current as a function of time during exposure to light (adapted from [54]).

Sharp voltage pulses of +5 V and −5 V are applied on the front and back gate, respectively, to induce injection barriers in the Si film (Figure 20a). Under a constant anode bias (V_A_ = 1 V), the device is exposed to light with intensity of 5 μW/cm^2^ and wavelength λ = 520 nm. Figure 20b shows the evolution of the potential barriers and anode current with the exposure time. The device is initially in the I_OFF_ state, due to the high potential barriers formed by the gate pulses. In the dark, the potential barrier height does not change appreciably in the tens of ms time scale, so that the device remains off. On the contrary, under illumination, the electrons photo-generated in the ungated region are attracted by the positively biased V_FG_ and accumulate under the top gate oxide. The accumulated photoelectrons reduce the injection barrier and V_ON_, which eventually switches the Z^2^–FET into the conduction mode with high I_ON_.

Two operation modes of the Z^2^–FET photodetector can be envisioned. Figure 20c shows the Z^2^–FET used as a switch triggered by a threshold light exposure. The device turns on after a few ms, with the delay arising from the time needed to photo-generate enough carriers to decrease the turn-on voltage and equal the reference anode voltage V_ON_ (*t*) = V_A_. The response time measures the light intensity.

**Figure 21:**
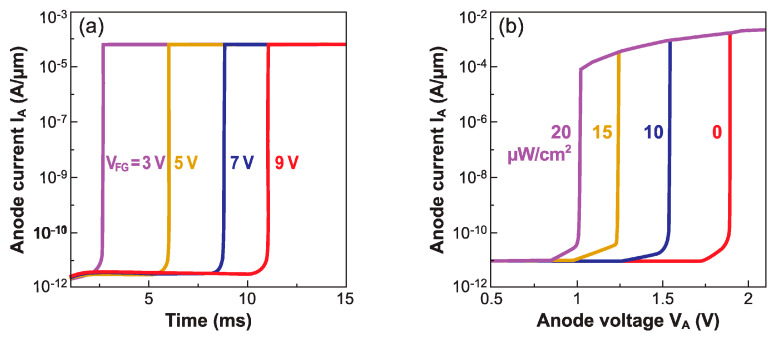
(**a**) Evolution of the anode current under exposure to continuous light for various gate voltages and V_A_ = 1 V. (**b**) Turn ON characteristics of the Z^2^–FET for various intensities of light pulse (adapted from [54,55]).

With a V_FG_ pulse, the non-equilibrium condition is reinforced and the potential barrier is taller. Hence, for constant light intensity, a longer exposure is needed to generate enough photoelectrons to lower the barrier and trigger the Z^2^–FET. For low V_FG_ bias, fewer photoelectrons are required and the response is faster. Figure 21a shows how the response time can be flexibly tuned by the gate voltage.

In an alternative operation mode, the Z^2^–FET is used as a sensor to monitor the light intensity. Once the gates are sharply pulsed into deep depletion, a short light flash with a constant duration (5 ms) generates photocarriers that lower the potential barrier and V_ON_. The anode voltage is ramped from 0 V up to 2.5 V and the current is monitored to detect the switching voltage (see Figure 21b). With higher light intensity, more photoelectrons are available and the V_ON_ reduction is more pronounced. Thus, a lower V_A_ is needed to turn on the device. The relation between exposure dose and V_ON_ is nearly linear, which facilitates the light-to-voltage conversion. Not only does the Z^2^–FET detect light, but it also integrates the photogenerated charge, that is the number of photons, and provides exceptional amplification through the positive feedback mechanism. These two operation modes have multiple potential applications.

### 5.5. Bio-Sensing

**Figure 22:**
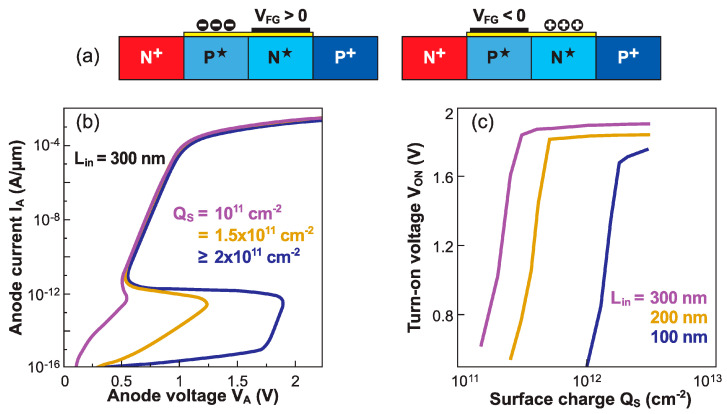
(**a**) Configuration of the Z^2^–FET operated as a biosensor for detection of negative and positive surface charges. (**b**) Output characteristics and (**c**) turn-on voltage vs. negative surface charge in devices with variable ungated region lengths.

The generic *NPNP* structure and overall functionality of a band-modulation device can be achieved by omitting the back-gate action and instead using surface charges attached to the ungated region (Figure 22a). Negative surface charges would induce the necessary *P*-type electrostatic doping that, together with V_FG_ > 0, gives rise to *S*-shaped characteristics. The sensing parameter is again the turn-on voltage V_ON_, which is highly dependent on the amount of surface charge. In Figure 22b, a very small variation in charge density (10^11^ cm^−2^) leads to a shift of V_ON_ as large as 1 V, showing outstanding detection capability.

The region of maximum sensitivity can be adjusted by modifying the length of the ungated region: *long* for small charge concentrations, or *short* for higher concentrations (Figure 22b). A complementary device, able to sense positive charges, can be designed by relocating the gate next to the cathode (right-hand scheme in Figure 22a). With V_FG_ < 0, the NPNP configuration is preserved.

## 6. Reconfigurable Sharp-Switching Modes

The mechanisms addressed in this section differ from band modulation. However, they can also provide sharp-switching capability and take place in the very same structure. These aspects emphasize the extraordinary versatility of the Z^2^–FET for reconfigurable circuits.

### 6.1. Impact Ionization MOSFET (I-MOS)

**Figure 23:**
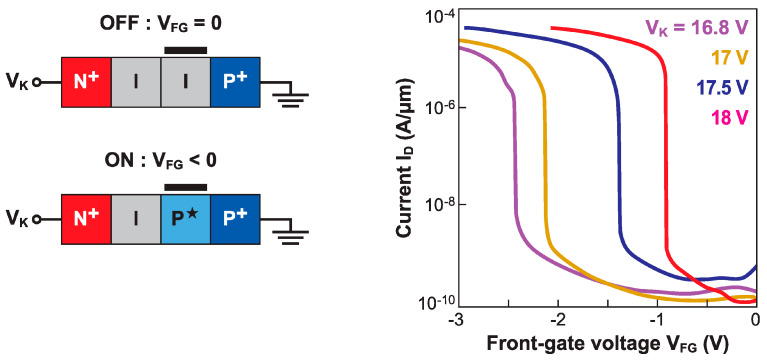
Current vs. gate voltage measured in a *P*-type I-MOS fabricated on FD-SOI (adapted from [57]).

The Z^2^–FET in Figure 2a can also operate as a *P*-type I-MOS. The difference is twofold: (i) the I-MOS looks like a PIN diode, rather than a virtual thyristor, and (ii) it is biased in reverse mode. In Figure 23, the gate voltage is negative, the anode is grounded, and the cathode voltage is large and positive, so as to trigger impact ionization. The *N*-type version would have the gate located nearby to the cathode and the voltage polarities swapped (V_FG_ > 0, V_A_ < 0) [56].

The principle is that in the off-state (V_FG_ = 0) the entire body is fully depleted and the two terminals are too far apart for impact ionization, resulting in a negligible current. A negative gate voltage starts bringing holes to the gated region. Such electrostatic doping virtually expands the anode contact underneath the gate, shortening the depleted region of the body. Even with V_K_ constant, the lateral electric field increases abruptly, initiating impact ionization in the ungated region. In other words, the I-MOS operates in the avalanche breakdown regime. The current turns on sharply, with an impressive subthreshold slope of 2 mV/decade [57].

The transfer characteristics reproduced in Figure 23 exhibit a large lateral shift for small variations in V_K_. The reason for this is the exponential dependence of the impact ionization rate on the electric field. An acceptable I_ON_/I_OFF_ current ratio (∼10^5^) is measured. Despite the very sharp switching and attractive threshold voltage (tunable via the cathode bias to less than 1 V), the I-MOS is ineligible for low-voltage applications. The cathode voltage is simply excessive, even when the gated and ungated regions are scaled down. The fundamental issue is that energy conservation during the impact ionization process imposes a minimum breakdown voltage of 3–4 *E*_G_ ~ 3.5–4.5 V in silicon [58]. Semiconductors with a narrower bandgap *E*_G_ (Ge, SiGe with a large Ge content, InGaAs, etc.) can provide a lower operating voltage [59], but they are also subject to high BTBT currents, especially when the impact ionization region is scaled down. This leads to high and exponentially increasing I_OFF_, dooming the I-MOS as being a low-power sharp-switching device.

Furthermore, like most avalanche-based devices, the I-MOS suffers from long switching times. Another major inconvenience results from the high carrier energy: hot electrons and holes injected into the gate dielectric and BOX are responsible for premature device aging.

### 6.2. TFET

**Figure 24:**
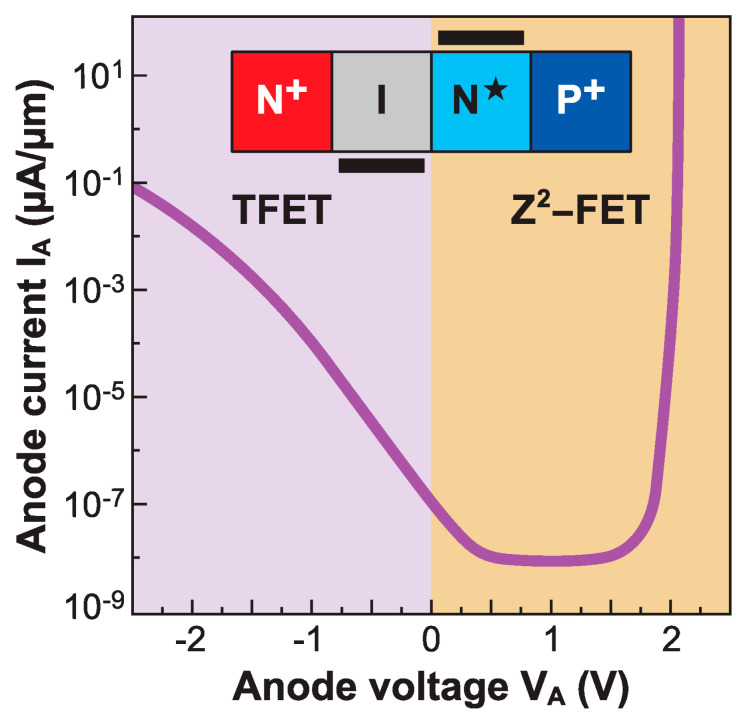
Experimental current vs. anode voltage characteristics showing the same device operating as a TFET (for V_A_ < 0) and a Z^2^–FET (for V_A_ > 0, adapted from [12]).

A band-modulation device can also be operated in the TFET mode, without any structural changes; the device anatomy and gate voltage polarity are preserved, but the anode is reverse biased. Figure 24 again shows a hocus-pocus diode with a positive V_FG_ > 0, a floating V_BG_ (no back-gate voltage), and a variable anode voltage V_A_. The I_A_–V_A_ characteristics are highly asymmetric, denoting fundamentally different operation mechanisms. When V_A_ is positive, we obtain the sharp-switching feedback-based Z^2^–FET characteristic described earlier, with a nearly vertical transition from I_ON_ to I_OFF_. On the other hand, the negative V_A_ produces a TFET mode, where the I_A_ is generated via band-to-band tunneling (BTBT) at the anode junction and flows between the heavily *P*^+^ anode and the electrostatically doped electron-rich region under the front gate.

The TFET mode of the band modulation device in Figure 24 has the merit of emphasizing the extreme reconfigurability of the band-modulation device, but the SS obtained experimentally is disappointing, as is the low I_ON_. These drawbacks are common to all experimentally realized Si-based TFETs. While numerical simulations relying on ideally abrupt junctions (and favorable adjustable parameters in the interband tunneling transmission coefficients) have led to predictions of SS below the 60 mV/decade limit over a wide range of current densities, including practically relevant I_ON_ [60], the reality is rather different. Due to the large Si bandgap and relatively heavy tunneling masses, no all-silicon TFET has delivered an I_ON_ exceeding 1 µA/µm, while maintaining sharp SS over several decades of I_D_. Attempts to improve I_ON_ by moving to lower E_G_ channel materials or by employing heterostructures, where the BTBT process would occur in a lower bandgap material (like Ge), while retaining CMOS compatibility have met with limited success [61,62].

**Figure 25:**
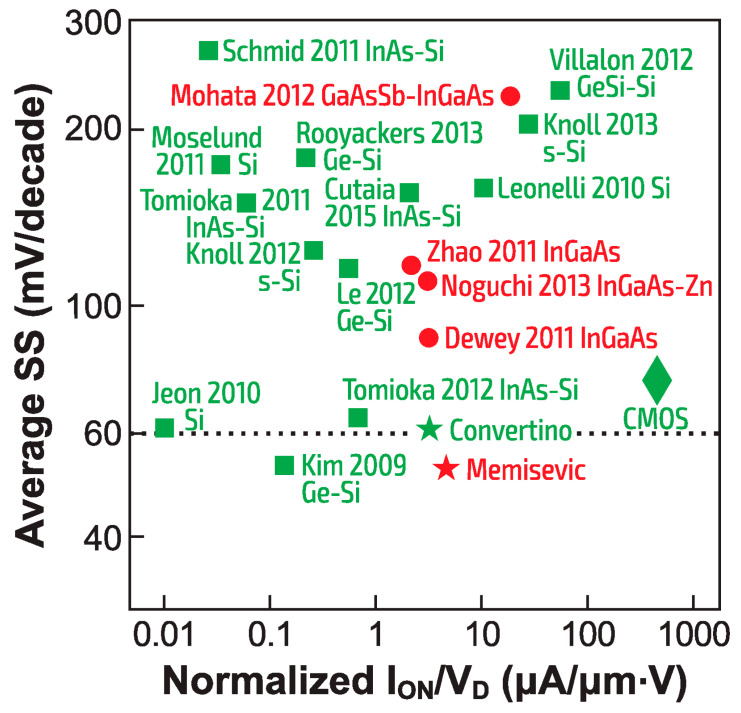
Average subthreshold swing SS vs. normalized I_ON_ plot, using the Cutaia et al. benchmarking approach [63]. Reproduced from [7], with additional recent data points from [64,65] indicated with stars. Green points indicate CMOS-compatible materials.

Even moving further afield from Si, experimentally realized TFETs with III-V channels have not provided a compelling combination of I_ON_ and low SS over a reasonable number of I_D_ decades. A comparison of TFET performance with room-temperature CMOS is provided in Figure 25, using the simple benchmarking technique proposed by Cutaia and co-workers [63]. The transfer characteristic at a fixed source–drain (i.e., anode–cathode) voltage V_D_ is analyzed by finding the average SS between I_OFF_ and I_ON_, which is plotted against I_ON_/V_D_. The lower-right corner, with SS slightly above the 60 mV/decade value and I_ON_ higher than 100 µm/(µm.V) is the domain of modern FinFET or FD-SOI CMOS transistors. Despite persistent efforts by researchers in a number of academic and industrial laboratories, including a recent tour-de-force of hybrid co-integration of III-V TFETs and MOSFETs on Si [64], no TFET to date has come close to outperforming CMOS at digital switching. This rather disappointing performance of the experimentally reported TFETs to date may spur the development of alternative CMOS-compatible sharp-switching devices, such as the band-modulation devices described in this article. One solution however for TFETs might consist in using the bipolar-enhanced TFET structure known as the BET-FET [66]; the tunneling current forms the base current and is naturally amplified by the internal gain of the bipolar transistor.

### 6.3. Esaki Diode

Esaki tunneling diodes [67] feature a negative differential resistance (NDR), attractive for fast-switching circuits [68] and high-frequency detectors and oscillators. The forward current is carried by the electrons tunneling from the conduction band on the *N*-side into the empty states of the valence band of the *P*-side. This mechanism is the reciprocal of the Zener effect.

BTBT occurs if the conduction and valence bands are properly aligned and the tunneling barrier is as thin as possible. These conditions imply the processing of a very sharp junction between heavily doped N and P regions, which is hard to achieve in ultrathin bodies. Another fundamental aspect that hampers tunneling in Si is the tunneling rate, exponentially higher in materials with lower bandgap *E*_G_ and effective mass.

While physical doping with degenerate concentration and sharp junctions is hardly feasible in FD-SOI, electrostatic doping can offer an attractive substitution. The device is biased such as to emulate a virtual *P*–*N* diode with the junction located at the left corner of the gate. High voltages applied to the front and back gates induce adjacent populations of free electrons and holes, with concentrations above 10^19^ cm^−3^. Although the depletion region is depth-dependent, it does not exceed 5–10 nm length. To avoid damaging the gate dielectric, the anode is grounded and the cathode is negatively biased; the BOX being much thicker, it can sustain high electric field without reliability concerns.

**Figure 26:**
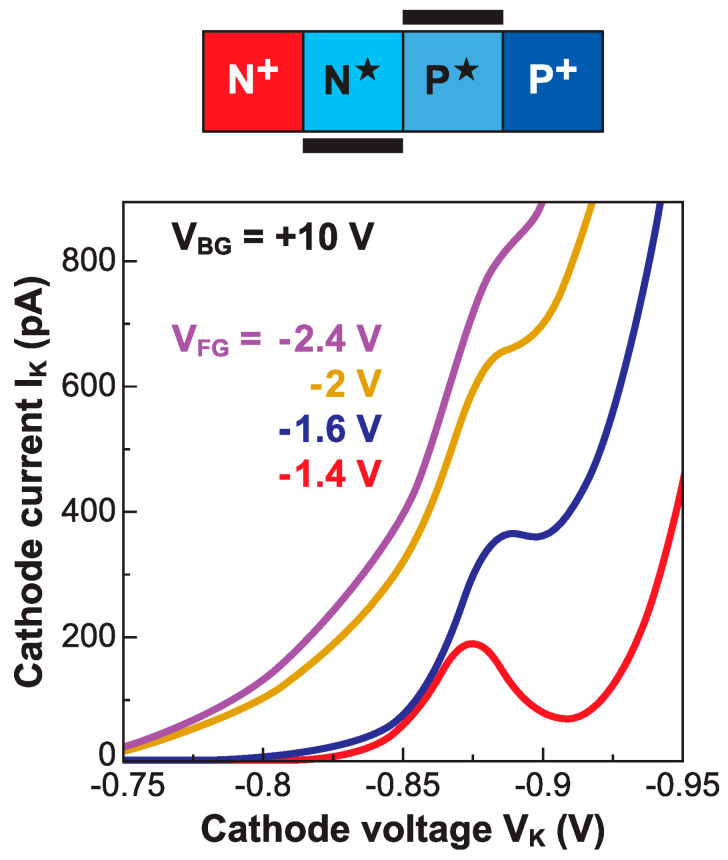
Experimental current–voltage characteristics of an electrostatic Esaki diode fabricated on undoped FD-SOI, showing the role of the gate voltage; with the same device parameters as in Figure 4 (adapted from [69]).

Measured at low temperature, where the generation current at interfaces is massively attenuated, the electrostatic Esaki diode exhibits a clear NDR region (Figure 26) [69]. The initial current is due to band-to-band tunneling and increases with cathode voltage. A peak current is reached before the tunneling current decays (for |V_K_| > 0.88 V). Then, the regular diffusion current of the *P*–*N* diode takes over. The NDR peak-to-valley ratio attains 3.6, a record in our silicon world and close to the best values in Si-SiGe heterojunctions [68]. Interestingly, 2D numerical simulations indicate that BTBT occurs at the front and back interfaces, as well as diagonally between the heavily-doped layers of electrons at the film–BOX interface and holes at the front-gate interface.

It is worth noting that for low gate voltage (|V_FG_| < 0.4 V), the concentration of holes P* on the anode side is too small to enable interband tunneling. On the other hand, at high V_FG_ the tunneling current increases and gives rise to a plateau that replaces the NDR peak (for –2 < *V*_FG_ < −1.6 V in Figure 26). This excess current originates from trap-assisted tunneling [69]. The electrons can tunnel either from the conduction band into empty deep traps located in the forbidden gap of the anode or from filled traps into the valence band of the cathode. Similarly, increasing the back-gate voltage is beneficial to tunneling, as long as the hole concentration set by the front gate is not affected. The solution, to avoid this undesirable coupling between the gates, is a virtual diode featuring twin ground-planes, as shown in Figure 3a. The *P*-type ground-plane must be located beneath the gated region and negatively biased to reinforce, rather than dilute, the P* electrostatic doping. This biasing scheme is important for keeping the junction between the two ground-planes reverse-biased and minimizing the leakage current.

These preliminary results emphasize the tremendous potential of electrostatic doping: an Esaki diode with a priori degenerate doping can actually be emulated in an undoped body using the front and back gates. Transferring this principle from FD-SOI to SiGe or pure Ge would certainly enhance the device performance.

## 7. Conclusions

The band-modulation mechanism was discovered thanks to the advent of high-quality ultrathin silicon films in FD-SOI technology. Without an ultrathin body, the necessary voltage-controlled blocking barriers could not be maintained across the whole film thickness. Given an insufficiently tall barrier at one of the interfaces, the regular forward current of the PIN diode would undermine the positive feedback mechanism and the excellent sharp-switching capability.

We do not know of any other device that can challenge the Z^2^–FET for steep switching (1 mV/decade) at low voltage (<1 V). Ferroelectric or tunneling FETs are unlikely to ever achieve this level of performance. The simplicity of the integration scheme, fully transferable from FD-SOI to FinFETs and nanowire transistors, is a strong argument for further development. Another important merit of band-modulation devices is their reconfigurability. For example, the Z^2^–RAM stands as the best option for embedded capacitorless 1T-DRAM memory. On the other hand, the Z^2^–FETs can protect their own circuits from electrostatic discharges.

The gate length can be scaled down, as in any advanced FD-SOI transistor. However, the presence of the ungated region is an inevitable obstacle to reaching less than twice the minimum length of a MOSFET.

While Z^2^–FETs can perform most of the key electronic functions (logic switching, memory, sensing, and ESD protection), they are still diodes in a world dominated by MOS transistors. Whether diodes have a chance to compete against, or work alongside, MOSFETs is a matter for future studies focusing on their reliability, variability, and downscaling.

## References

[1] See the 2021 International Roadmap for Devices and Systems, Specifically the More Moore Section. https://irds.ieee.org/images/files/pdf/2021/2021IRDS_MM.pdf.

[2] Taur Y., Ning T.H. (2021). Fundamentals of Modern VLSI Devices.

[3] Cristoloveanu S. (2021). Fully Depleted Silicon-On-Insulator: Nanodevices, Mechanisms and Characterization.

[4] Beckers A., Jazaeri F., Enz C. (2018). Characterization and Modeling of 28-nm Bulk CMOS Technology Down to 4.2 K. IEEE J. Electron Devices Soc..

[5] Incandela R.M., Song L., Homulle H., Charbon E., Vladimirescu A., Sebastiano F. (2018). Characterization and compact modeling of nanometer CMOS transistors at deep-cryogenic temperatures. J. Electron Device Soc..

[6] Zaslavsky A., Richter C.A., Shrestha P.R., Hoskins B.D., Le S.T., Madhavan A., McClelland J.M. (2021). Impact ionization-induced bistability in CMOS transistors at cryogenic temperatures for capacitorless memory applications. Appl. Phys. Lett..

[7] Cristoloveanu S., Wan J., Zaslavsky A. (2016). A review of sharp-switching devices for ultra-low power applications. J. Electron Device Soc..

[8] Antonov R.D., Johnson A.T. (1999). Subband Population in a Single-Wall Carbon Nanotube Diode. Phys. Rev. Lett..

[9] Hueting R.J.E., Rajasekharan B., Salm C., Schmitz J. (2008). The Charge Plasma P-N Diode. IEEE Electron Device Lett..

[10] Heinzig A., Slesazeck S., Kreupl F., Mikolajick T., Weber W.M. (2011). Reconfigurable Silicon Nanowire Transistors. Nano Lett..

[11] Cristoloveanu S., Lee K.H., Park H., Parihar M.S. (2019). The concept of electrostatic doping and related devices. Solid-State Electron..

[12] Cristoloveanu S., Lee K.H., Bawedin M. (2017). A reconfigurable silicon-on-insulator diode with tunable electrostatic doping. J. Appl. Phys..

[13] Wan J., Le Royer C., Zaslavsky A., Cristoloveanu S. (2011). A Compact Capacitor-Less High-Speed DRAM Using Field Effect-Controlled Charge Regeneration. IEEE Electron Device Lett..

[14] Wan J., Cristoloveanu S., Le Royer C., Zaslavsky A. (2012). A feedback silicon-on-insulator steep switching device with gate-controlled carrier injection. Solid-State Electron..

[15] Sheikhian I., Raissi F. (2003). High-speed digital family using field effect diode. Electron. Lett..

[16] Salman A.A., Beebe S.G., Emam M., Pelella M.M., Ioannou D.E. Field Effect Diode (FED): A novel device for ESD protection in deep sub-micron SOI technologies. Proceedings of the IEEE International Electron Devices Meeting (IEDM).

[17] El Dirani H., Fonteneau P., Solaro Y., Ferrari P., Cristoloveanu S. Novel FDSOI band-modulation device: Z^2^-FET with dual ground planes. Proceedings of the 46th European Solid-State Device Research Conference (ESSDERC’2016).

[18] Solaro Y., Fonteneau P., Legrand C.-A., Marin-Cudraz D., Passieux J., Guyader P., Clement L.-R., Fenouillet-Beranger C., Ferrari P., Cristoloveanu S. Innovative ESD protections for UTBB FD-SOI technology. Proceedings of the IEEE International Electron Devices Meeting (IEDM).

[19] Solaro Y., Fonteneau P., Legrand C., Fenouillet-Beranger C., Ferrari P., Cristoloveanu S. (2015). A sharp-switching device with free surface and buried gates based on band modulation and feedback mechanisms. Solid-State Electron..

[20] Weber O., Josse E., Andrieu F., Cros A., Richard E., Perreau P., Baylac E., Degors N., Gallon C., Perrin E. 14nm FDSOI technology for high speed and energy efficient applications. Proceedings of the 2014 Symposium on VLSI Technology (VLSIT).

[21] Planes N., Weber O., Barral V., Haendler S., Noblet D., Croain D., Bocat M., Sassoulas P.-O., Federspiel X., Cros A. 28nm FDSOI technology platform for high-speed low-voltage digital applications. Proceedings of the 2012 Symposium on VLSI Technology (VLSIT).

[22] Cristoloveanu S., Lee K., Parihar M., El Dirani H., Lacord J., Martinie S., Le Royer C., Barbe J.-C., Mescot X., Fonteneau P. (2018). A review of the Z^2^ -FET 1T-DRAM memory: Operation mechanisms and key parameters. Solid-State Electron..

[23] Cristoloveanu S., Athanasiou S., Bawedin M., Galy P. (2017). Evidence of Supercoupling Effect in Ultrathin Silicon Layers Using a Four-Gate MOSFET. IEEE Electron Device Lett..

[24] Navarro C., Bawedin M., Andrieu F., Sagnes B., Martinez F., Cristoloveanu S. (2015). Supercoupling effect in short-channel ultrathin fully depleted silicon-on-insulator transistors. J. Appl. Phys..

[25] Parihar M.S., Lee K.H., Park H.J., Lacord J., Martinie S., Barbé J.C., Xu Y., El Dirani H., Taur Y., Cristoloveanu S. (2018). Insight into carrier lifetime impact on band-modulation devices. Solid-State Electron..

[26] Wan J., Cristoloveanu S., le Royer C., Zaslavsky A. (2013). A systematic study of the sharp-switching Z^2^-FET device: From mechanism to modeling and compact memory applications. Solid-State Electron..

[27] El Dirani H., Solaro Y., Fonteneau P., Legrand C.-A., Marin-Cudraz D., Golanski D., Ferrari P., Cristoloveanu S. (2016). A band-modulation device in advanced FDSOI technology: Sharp switching characteristics. Solid-State Electron..

[28] Taur Y., Lacord J., Parihar M.S., Wan J., Martinie S., Lee K., Bawedin M., Barbe J.-C., Cristoloveanu S. (2017). A comprehensive model on field-effect pnpn devices (Z^2^-FET). Solid-State Electron..

[29] Martinie S., Lacord J., Lee K., Bawedin M., Cristoloveanu S. (2021). Pragmatic Z^2^-FET compact model including DC and 1T-DRAM memory operation. Solid-State Electron..

[30] Gatard E. (2006). Analyse des Phénomènes Physiques dans les Diodes p-i-n: Contribution à la Modélisation Électrothermique pour les Applications de Puissance RF et Hyperfréquences. Ph.D. Thesis.

[31] Rozeau O., Jaud M.-A., Poiroux T., Benosman M. Surface potential based model of ultra-thin fully depleted SOI MOSFET for IC simulations. Proceedings of the SOI Conference (SOI).

[32] Lee K.-H., El Dirani H., Fonteneau P., Bawedin M., Cristoloveanu S. (2019). Sharp Logic Switch Based on Band Modulation. IEEE Electron Device Lett..

[33] El Dirani H., Solaro Y., Fonteneau P., Ferrari P., Cristoloveanu S. (2016). Properties and mechanisms of Z^2^-FET at variable temperature. Solid-State Electron..

[34] Navarro C., Lacord J., Parihar M.S., Adamu-Lema F., Duan M., Rodriguez N., Cheng B., El Dirani H., Barbe J.C., Fonteneau P. (2017). Extended analysis of the Z^2^-FET: Operation as capacitorless eDRAM. IEEE Trans. Electron. Devices.

[35] Bawedin M., Cristoloveanu S., Flandre D., Udrea F. (2010). Dynamic body potential variation in FD SOI MOSFETs operated in deep non-equilibrium regime: Model and applications. Solid-State Electron..

[36] Bawedin M., Cristoloveanu S., Hubert A., Park K.-H., Martinez F. (2011). Floating Body SOI Memory: The Scaling Tournament. Semiconductor-On-Insulator Materials for Nanoelectronics Applications.

[37] Lacord J., Parihar M.S., Navarro C., Wakam F.T., Bawedin M., Cristoloveanu S., Gamiz F., Barbe J.-C. MSDRAM, A2RAM and Z^2^-FET performance benchmark for 1T-DRAM applications. Proceedings of the 2018 International Conference on Simulation of Semiconductor Processes and Devices (SISPAD).

[38] El Dirani H., Lee K.H., Parihar M.S., Lacord J., Martinie S., Barbe J.C., Mescot X., Fonteneau P., Broquin J.E., Ghibaudo G. (2017). Ultra-low power 1T-DRAM in FDSOI technology. Microelectron. Eng..

[39] Marquez C., Navarro S., Navarro C., Salazar N., Galy P., Cristoloveanu S., Gamiz F. Temperature and gate leakage influence on the Z^2^-FET memory operation. Proceedings of the 49th European Solid-State Device Research Conference (ESSDERC).

[40] Duan M., Navarro C., Cheng B., Adamu-Lema F., Wang X., Georgiev V.P., Gamiz F., Millar C., Asenov A. (2019). Thorough understanding of retention time of Z^2^-FET memory operation. IEEE Trans. Electron. Devices.

[41] Kwon S., Navarro C., Gamiz F., Galy P., Cristoloveanu S., Kim Y.-T., Ahn J. (2021). Improved Retention Characteristics of Z^2^-FET Employing Half Back-Gate Control. IEEE Trans. Electron Devices.

[42] Parihar M.S., Lee K.H., El Dirani H., Navarro C., Lacord J., Martinie S., Barbe J.-C., Fonteneau P., Galy P., Le Royer C. Low-power Z^2^-FET capacitorless 1T-DRAM. Proceedings of the International Memory Workshop.

[43] Navarro C., Marquez C., Navarro S., Lozano C., Kwon S., Kim Y.-T., Gamiz F. (2019). Simulation Perspectives of Sub-1V Single-Supply Z^2^-FET 1T-DRAM Cells for Low-Power. IEEE Access.

[44] Navarro S., Navarro C., Marquez C., Salazar N., Galy P., Cristoloveanu S., Gamiz F. (2019). Reliability Study of Thin-Oxide Zero-Ionization, Zero-Swing FET 1T-DRAM Memory Cell. IEEE Electron Device Lett..

[45] CMarquez C., Navarro C., Navarro S., Padilla J.L., Donetti L., Sampedro C., Galy P., Kim Y.-T., Gamiz F. (2019). On the Low-Frequency Noise Characterization of Z^2^-FET Devices. IEEE Access.

[46] Adamu-Lema F., Duan M., Georgiev V., Asenov P.A. A carrier lifetime sensitivity probe based on transient capacitance: A novel method to characterize lifetime in Z^2^-FET. Proceedings of the International Conference on Simulation of Semiconductor Processes and Devices, SISPAD.

[47] Navarro C., Navarro S., Marquez C., Padilla J.L., Galy P., Gamiz F. (2019). 3-D TCAD Study of the Implications of Channel Width and Interface States on FD-SOI Z^2^-FETs. IEEE Trans. Electron Devices.

[48] Navarro C., Duan M., Parihar M.S., Adamu-Lema F., Coseman S., Lacord J., Lee K., Sampedro C., Cheng B., El Dirani H. (2017). Z^2^-FET as capacitor-less eDRAM cell for high-density integration. IEEE Trans. Electron Devices.

[49] Wan J., Le Royer C., Zaslavsky A., Cristoloveanu S. (2013). Progress in Z^2^-FET 1T-DRAM: Retention time, writing modes, selective array operation, and dual bit storage. Solid-State Electron..

[50] Navarro S., Navarro C., Marquez C., El Dirani H., Galy P., Bawedin M., Pickering A., Cristoloveanu S., Gamiz F. (2018). Experimental Demonstration of Operational Z^2^-FET Memory Matrix. IEEE Electron Device Lett..

[51] Kwon S., Navarro C., Galy P., Cristoloveanu S., Gamiz F., Ahn J., Kim Y.T. (2020). Operations of zero impact ionization, zero subthreshold swing FET matrix without selectors. IEEE Electron Device Lett..

[52] Kwon S., Navarro C., Gamiz F., Cristoloveanu S., Kim Y.-T., Ahn J. (2021). Memory Operation of Z^2^-FET Without Selector at High Temperature. IEEE J. Electron Devices Soc..

[53] Solaro Y., Fonteneau P., Legrand C.A., Marin-Cudraz D., Passieux J., Guyader P., Clement L.R., Fenouillet-Beranger C., Ferrari P., Cristoloveanu S. Thin body ESD protections in 28nm UTBB-FDSOI: From static to transient behavior. Proceedings of the Electrical Overstress/Electrostatic Discharge Symposium (EOS/ESD).

[54] Liu J., Cao X.Y., Lu B.R., Chen Y.F., Zaslavsky A., Cristoloveanu S., Wan J. (2019). Dynamic coupling effect in Z^2^-FET and its application for photodetection. IEEE J. Electron Devices Soc..

[55] Liu J., Cao X.Y., Lu B.R., Chen Y.F., Zaslavsky A., Cristoloveanu S., Bawedin M., Wan J. A new photodetector on SOI. Proceedings of the IEEE SOI-3D-Subthreshold Microelectronics Technology Unified Conference (S3S).

[56] Gopalakrishnan K., Griffin P., Plummer J. I-MOS: A novel semiconductor device with a subthreshold slope lower than kT/q. Proceedings of the Tech. Dig. IEEE International Electron Devices Meeting (IEDM).

[57] Mayer F., le Royer C., le Carval G., Tabone C., Clavelier L., Deleonibus S. (2007). Comparative study of the fabricated and simulated impact ionization MOS (IMOS). Solid-State Electron..

[58] Savio A., Monfray S., Charbuillet C., Skotnicki T. (2009). On the Limitations of Silicon for I-MOS Integration. IEEE Trans. Electron Devices.

[59] Sarkar D., Singh N., Banerjee K. (2010). A Novel Enhanced Electric-Field Impact-Ionization MOS Transistor. IEEE Electron Device Lett..

[60] Lu H., Seabaugh A. (2014). Tunnel Field-Effect Transistors: State-of-the-Art. J. Electron Devices Soc..

[61] Kim S.H., Kam H., Hu C., Liu T.-J.K. Germanium-source tunnel field effect transistors with record high I_ON_/I_OFF_. Proceedings of the Symposium on VLSI Technology.

[62] Le S.T., Jannaty P., Luo X., Zaslavsky A., Perea D.E., Dayeh S., Picraux S.T. (2012). Axial SiGe Heteronanowire Tunneling Field-Effect Transistors. Nano Lett..

[63] Cutaia D., Moselund K.E., Borg M., Schmid H., Gignac L., Breslin C.M., Karg S., Uccelli E., Riel H. (2015). Vertical InAs-Si gate-all-around tunnel FETs integrated on Si using selective epitaxy in nanotube templates. IEEE J. Electron Devices Soc..

[64] Convertino C., Zota C.B., Schmid H., Caimi D., Czornomaz L., Ionescu A.M., Moselund K.E. (2021). A hybrid III–V tunnel FET and MOSFET technology platform integrated on silicon. Nat. Electron..

[65] Memisevic E., Svensson J., Hellenbrand M., Lind E., Wernersson L.-E. (2016). Vertical InAs/GaAsSb/GaSb tunneling field-effect transistor on Si with S = 48 mV/decade and Ion = 10 μA/μm for Ioff = 1 nA/μm at Vds = 0.3 V. IEEE IEDM.

[66] Wan J., Zaslavsky A., Le Royer C., Cristoloveanu S. (2013). Novel Bipolar-Enhanced Tunneling FET with Simulated High On-Current. IEEE Electron Device Lett..

[67] Esaki L. (1976). Discovery of the tunnel diode. IEEE Trans. Electron Devices.

[68] Berger P.R., Ramesh A. (2011). Negative Differential Resistance Devices and Circuits. Comprehensive Semiconductor Science Technology: Online Version.

[69] Lee K.-H., Cristoloveanu S. (2019). Esaki Diode in Undoped Silicon Film. IEEE Electron Device Lett..

